# A combination of Notch signaling, preferential adhesion and endocytosis induces a slow mode of cell intercalation in the *Drosophila* retina

**DOI:** 10.1242/dev.197301

**Published:** 2021-05-17

**Authors:** Laura Blackie, Melda Tozluoglu, Mateusz Trylinski, Rhian F. Walther, François Schweisguth, Yanlan Mao, Franck Pichaud

**Affiliations:** 1 MRC Laboratory for Molecular Cell Biology (LMCB), University College London, London WC1E 6BT, UK; 2 MRC London Institute of Medical Sciences (LMS), London W12 0NN, UK; 3 Department of Developmental and Stem Cell Biology, Pasteur Institute, F-75015 Paris, France; 4 CNRS, UMR3738, F-75015 Paris, France; 5 Institute for the Physics of Living Systems, University College London, London WC1E 6BT, UK

**Keywords:** Adherens junction, Adhesion, Cell intercalation, Epithelia, Nephrins, Notch

## Abstract

Movement of epithelial cells in a tissue occurs through neighbor exchange and drives tissue shape changes. It requires intercellular junction remodeling, a process typically powered by the contractile actomyosin cytoskeleton. This has been investigated mainly in homogeneous epithelia, where intercalation takes minutes. However, in some tissues, intercalation involves different cell types and can take hours. Whether slow and fast intercalation share the same mechanisms remains to be examined. To address this issue, we used the fly eye, where the cone cells exchange neighbors over ∼10 h to shape the lens. We uncovered three pathways regulating this slow mode of cell intercalation. First, we found a limited requirement for MyosinII. In this case, mathematical modeling predicts an adhesion-dominant intercalation mechanism. Genetic experiments support this prediction, revealing a role for adhesion through the Nephrin proteins Roughest and Hibris. Second, we found that cone cell intercalation is regulated by the Notch pathway. Third, we show that endocytosis is required for membrane removal and Notch activation. Taken together, our work indicates that adhesion, endocytosis and Notch can direct slow cell intercalation during tissue morphogenesis.

## Abstract (precis)

**Summary:** Cell intercalation via intercellular junction remodeling can occur over minutes or even hours. Three pathways, adhesion, endocytosis and Notch, were found to induce slow intercalation in cone cells of the fly retina.

## INTRODUCTION

Epithelial cells are polarized along the apical (top)-basal (bottom) axis and assemble into tissues via their lateral adherens junctions (AJs). Loss and creation of AJs between cells can shape tissues by rearranging the relative position of cells within the plane of the epithelium. For example, in the *Drosophila* germband, polarized steps of AJ loss and creation promote tissue elongation by inducing cell intercalation along the anterior-posterior (A/P) axis of the embryo (Bertet et al., 2004; Blankenship et al., 2006; Tetley et al., 2016; Zallen and Wieschaus, 2004). Similar regulations take place, for example, between mesodermal cells in zebrafish to promote convergent extension (Yin et al., 2008) or during renal tube development in *Xenopus* (Lienkamp et al., 2012). In these relatively homogeneous tissues, intercalation between groups of four cells takes place over minutes.

In the germband, the RhoA-Rok-MyosinII (MyoII) pathway controls actomyosin contractility, and E-cadherin (Ecad) mediates adhesion (Garcia De Las Bayonas et al., 2019; Levayer and Lecuit, 2013; Levayer et al., 2011; Munjal et al., 2015; Pare et al., 2014; Rauzi et al., 2010; Simões et al., 2010, 2014). Examining actomyosin dynamics in groups of four intercalating cells in this tissue shows that polarized actomyosin flows generate contractile forces that are harnessed by the junctional pool of actomyosin to remodel AJs between cells. Thus, MyoII accumulates preferentially at the AJs that shrink to drive intercalation. In this tissue, cell intercalation also requires endocytosis to shed membrane as an AJ is eliminated (Levayer et al., 2011). In other tissues, such as the fly notum and wing disc, where cell intercalation also occurs over minutes, neighbor exchange is stochastic and reversible. Here, although MyoII is required for intercalation, its excessive accumulation at the AJ inhibits this intercalation (Curran et al., 2017). Furthermore, in the dorsal branch of the fly trachea, where cells intercalate to form tubes, MyoII is largely dispensable. In this case, intercalation between branch cells is induced by forces that are generated by the migrating tip cell, which pulls on the branch cells (Ochoa-Espinosa et al., 2017). Importantly, these examples indicate that there is no simple relationship between MyoII and intercalation.

**Fig. 1.**
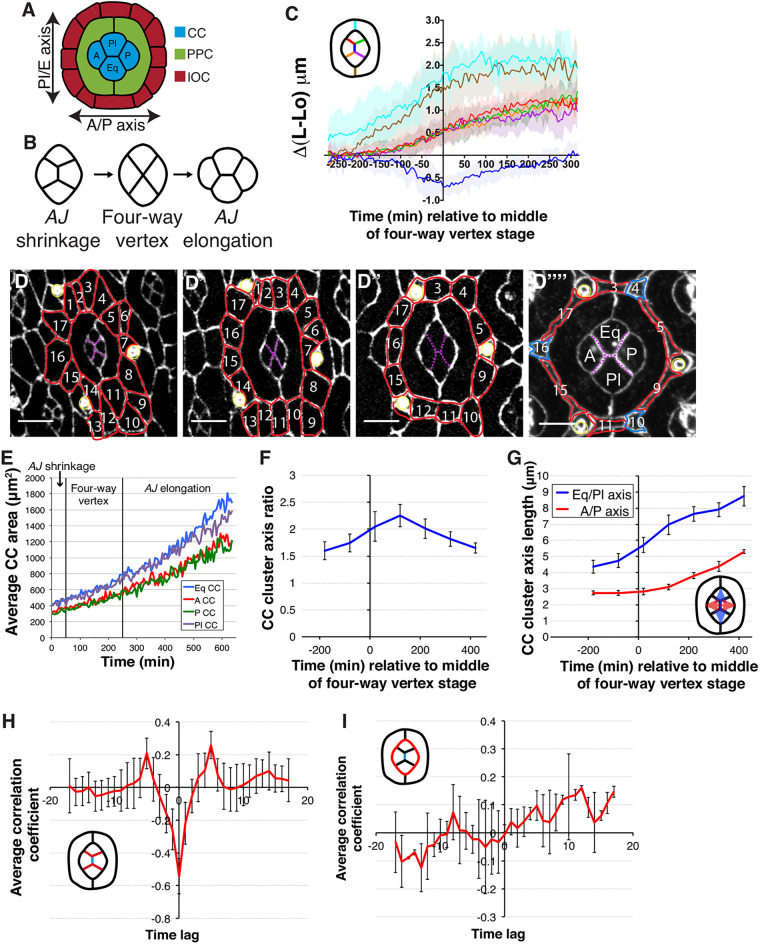
**Cone cell intercalation and retinal cell growth trajectories.** (A) The arrangement of cells in the ommatidium. A, anterior; CC, cone cell; Eq, equatorial; IOC, interommatidial cell; P, posterior; Pl, polar; PPC, primary pigment cell. (B) Stages of CC intercalation. (C) Average relative length (L-L0) of CC and PPC AJs during ommatidium development. Time 0 is the middle of the four-way vertex stage (*n*=13 ommatidia from two retinas). The different colored lines in the graph refer to the cell boundaries depicted in the schematic. (D) Confocal sections taken from a time-lapse movie of ommatidium development, with AJs labeled with endogenous Ecad::GFP. IOCs are outlined in red and numbered through subsequent frames. Tertiary pigment cells are labeled in blue. Dashed purple lines indicate the inter CC AJs and yellow outlines indicate the bristle cell complexes. (E) Average apical area of CCs over time (*n*=4 ommatidia). Vertical lines demarcate the stages of CC intercalation. (F) Average CC cluster axis ratio over time relative to the middle of the four-way vertex stage (*n*=14 ommatidia). (G) Average lengths of CC cluster axes over time relative to the middle of the four-way vertex stage (*n*=14 ommatidia). (H) Average cross-correlation of the rate of change in the length of the central CC AJ (shown in blue in the schematic), with the adjoining CC AJs (shown in red in the schematic). Correlation coefficient: r=-0.54±0.11 (mean±s.d.) at a time lag of 0 (*n*=13 ommatidia). (I) Average cross-correlation of rate of change in the length of the central CC AJ (shown in blue in the schematic) with the CC-PPC AJ (shown in red in the schematic) (*n*=13 ommatidia). Scale bars: 5 μm. Error bars indicate s.d. in F-I.

The fly ommatidium, which is the basic visual unit of the insect compound eye, presents an interesting departure point from all these tissues in that it includes a deterministic step of neighbor exchange between four cells that unfolds over ∼10 h and that is reproducible. This slow intercalation occurs during lens formation between four epithelial-like cells called cone cells. Whether this type of slow intercalation is governed by the same mechanisms that underpin faster intercalation has not been investigated in detail. In the ommatidium, the four core cone cells are surrounded by two large primary pigment cells, which are themselves surrounded by a complement of narrow secondary and tertiary pigment cells, collectively referred to as ‘interommatidial cells’ (Cagan, 2009; Cagan and Ready, 1989; Charlton-Perkins et al., 2017; Ready et al., 1976; Wolff and Ready, 1993) (Fig. 1A). As they are specified, the cone and pigment cells each find their location in the 2D plane of the lens through highly regulated steps of neighbor exchange (Cagan and Ready, 1989; Larson et al., 2008). These steps are controlled by preferential adhesion, whereby adhesion is favored between primary pigment cells and interommatidial precursors, and is minimized among interommatidial cells. This preferential adhesion relies on the Neph/Nephrin-like immunoglobulin adhesion protein family. Hibris (Hbs; Nephrin-like) is expressed in primary pigment cells and binds to Roughest (Rst; Neph-1), which is expressed in interommatidial precursors (Bao and Cagan, 2005). In addition, Hbs functions in the four core cone cells to regulate their intercalation (Grillo-Hill and Wolff, 2009), suggesting that preferential adhesion is involved in cone cell intercalation. However, no requirement for Rst has been found in these cells so far. In addition, Ncadherin (Ncad) has also been shown to play a role in regulating cone cell intercalation by establishing a planar polarized pattern of interfacial tension within the cone cell quartet. In this pattern, a higher tension, generated by MyoII, is found at the interface between the cone and primary pigment cells compared with the AJs between the cone cells, where MyoII tension is limited (Chan et al., 2017). However, it is unclear how this MyoII pattern contributes to regulating intercalation in these cells and how it relates to preferential adhesion through Hbs. In studying this issue, we found little requirement for RhoA-MyoII during cone cell intercalation. Instead, our results indicate a pre-eminent role for the conserved Notch signaling pathways, adhesion through both Hbs and Rst, and endocytosis. Taken together, our results suggest that preferential adhesion between cells is a principal mechanism of slow intercalation.

## RESULTS

### Adherens junction dynamics during cone cell intercalation

Cone cell intercalation unfolds over 10 h (Fig. 1A-C and Movie 1) and consists of the elimination of the AJ between the A/P cells, followed by the creation of a new AJ between the polar and equatorial (Pl/Eq) cells (Fig. 1B,C). To establish the cellular dynamics associated with this step of slow intercalation, we used the Ecad::GFP transgene to monitor how the AJs evolve as the ommatidium develops. We found that, as the cone cells undergo neighbor exchange, they also increase their apical area (Fig. 1D,E, Movie 1). Quantification revealed that, despite the elimination of the AJ between the A/P cells, the cone cell cluster elongates along the Pl/Eq axis upon intercalation (Fig. 1F,G). Following this, the cone cell quartet widens along the A/P axis as the AJ is created between the Pl/Eq cells. Furthermore, examining the dynamics of growth of the AJs shared by the four cone cells, we found a cross-correlation between the shrinkage of the A/P cone cell AJ and the expansion of the remaining adjoining AJs (Fig. 1H). Thus, a mechanism might exist whereby membrane removed from the shrinking AJ is recycled to the neighboring AJs. This correlation did not hold when considering the AJs shared with primary pigment cells by cone cells (Fig. 1I), suggesting local membrane redistribution between the AJs shared amongst the cone cells, but not with those shared by cone cells with primary pigment cells.

Next, we considered that the primary pigment cells that surround the cone cells could influence cone cell intercalation and shape. These two pigment cells share AJs that run parallel to the Pl/Eq axis of the lens and, thus, are aligned with the shrinking cone cell AJ (Fig. 1A). As the cone cell AJ shrinks, the AJs shared by the primary pigment cells lengthen (Fig. 1C,D), as demonstrated by the negative correlation of their length (Fig. S1A). Here, we reasoned that the negative correlation could indicate that these processes are linked. To test this idea, we compared the corresponding patterns of AJ length fluctuation by cross-correlating the fluctuation of the lengths of these AJs. However, quantification of this parameter showed that they were not correlated, suggesting that length changes in the primary pigment cell AJs do not directly influence changes in length of the cone cell AJs (Fig. S1B).

### Limited MyoII accumulation at the shrinking adherens junction suggests a minimal role in intercalation

**Fig. 2.**
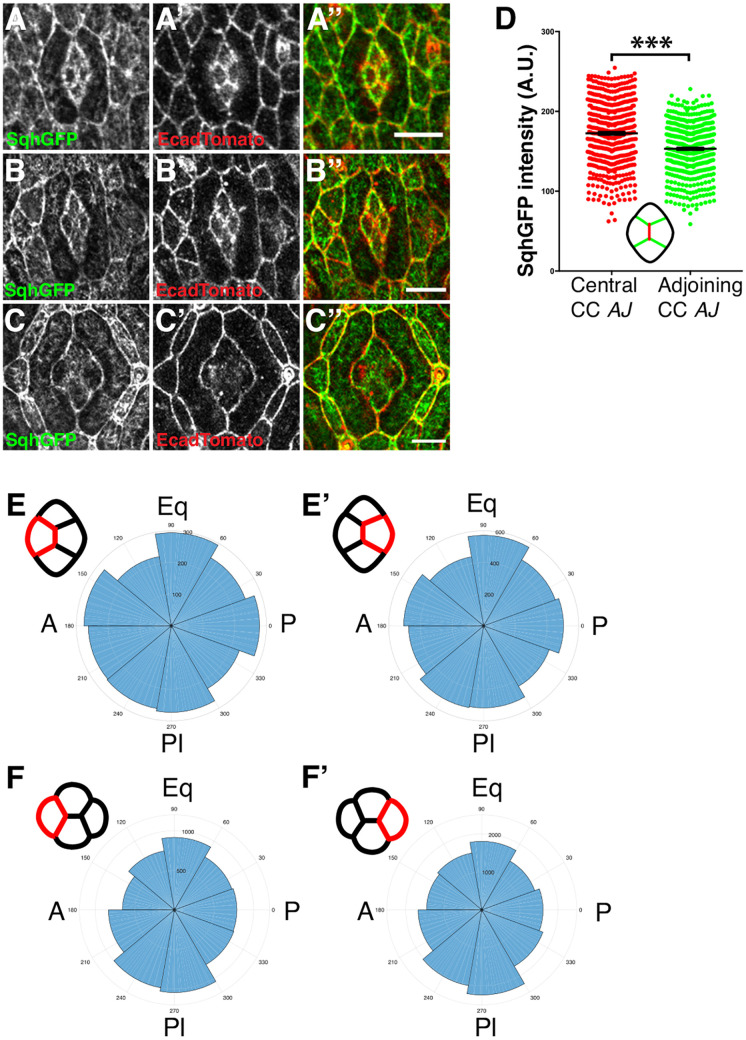
**MyoII expression and dynamics in cone cell intercalation.** (A-C) *sqh^AX3^;sqh-sqh::GFP/+;Ecad::Tomato/+* flies showing localization of MyoII (Sqh::GFP) at the junction shrinkage (A-A″), four-way vertex (B-B″) and junction elongation (C-C″) stages of cone cell (CC) intercalation. (D) Quantification at the AJ shrinkage stage of Sqh::GFP intensity on shrinking A/P cone cell AJs (red) compared with adjoining AJs (green) paired by ommatidium (*n*=514 ommatidia). Student's *t*-test: ***P<0.0001. Data are mean±s.e.m. (E,F′) Polar histograms showing directions of MyoII flow vectors calculated by PIV. (E) A and (E′) P cone cells during AJ shrinkage phase. (F) A and (F′) P cone cells during AJ elongation phase. Scale bars: 5 μm.

To assess whether MyoII powers AJ remodeling to induce cone cell intercalation, we analyzed its distribution and intensity over time using a fly strain where the Myosin light chain is tagged with GFP (Sqh::GFP) (Fig. 2A-C). We detected a marginal 10% MyoII enrichment at the shrinking AJ between the A/P cone cells (Fig. 2D). This was small compared with the values of 30-300% enrichment reported for shrinking AJs in the germband (Collinet et al., 2015; Pare et al., 2014; Simões et al., 2010). Using live imaging, we could also visualize pulsatile apical-medial meshworks of MyoII in the cone cells; however, unlike in the intercalating cells in the germband, we could not detect any flow dynamics toward the shrinking AJ (Fig. 2E) or later on towards the new AJ as it was created between the Pl/Eq cone cells (Fig. 2F). Taken together, these results suggest that slow cell intercalation in the eye lens is not driven primarily by MyoII contractility.

### Modeling cone cell intercalation predicts a predominant role for adhesion

Although the small increase in Sqh::GFP accumulation at the A/P AJ compared with the adjoining AJs could regulate shrinkage of this AJ, its small magnitude could mean that this MyoII activity is not sufficient to drive AJ shrinkage and that adhesion between cells might dominate this process instead. It is also possible that extrinsic regulations are involved, for example from the primary pigment cells that surround the cone cells. To distinguish between these possibilities and assess a potential role for the primary pigment cells during cone cell intercalation, we built a computational vertex model (Farhadifar et al., 2007) of the ommatidium, allowing us to vary the relative levels of contractility and adhesion at the AJs of the cells.

**Fig. 3.**
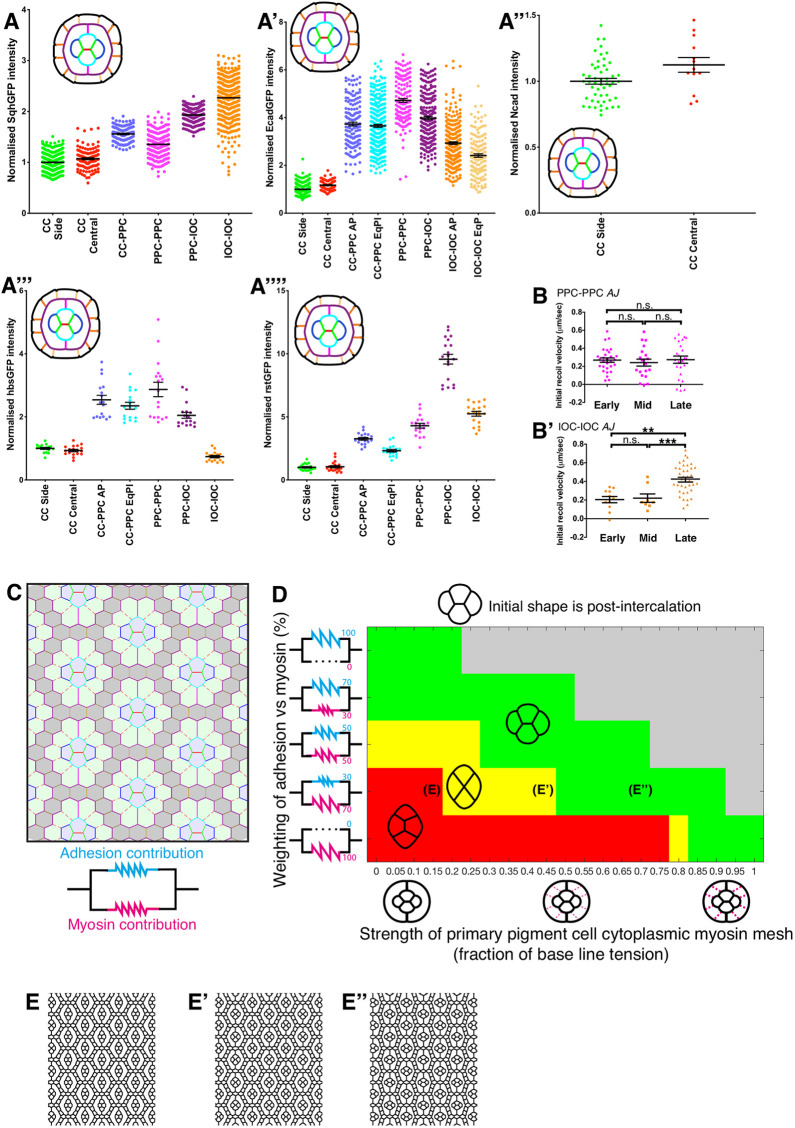
**Modeling the contribution of MyoII contractility and adhesion to cone cell intercalation.** (A-A″″) Quantification at the AJ elongation stage of (A) Sqh::GFP, (A′) Ecad::GFP, (A″) Ncad staining, (A‴) Hbs::GFP intensity and (A″″) Rst::GFP intensity on each AJ type normalized to the average of the cone cell-cone cell-side AJ (shown in green). CC, cone cell; IOC, interommatidial cell; PPC, primary pigment cell. Data are mean±s.e.m. (B,B′) Initial recoil velocity of ablation of (B) PPC-PPC AJs and (B′) IOC-IOC AJs at each stage of ommatidial development. For PPC-PPC AJs: one-way ANOVA n.s. *P*=0.784, *n*=29, 20 and 24 AJs for early, mid and late stages of development, respectively. For IOC-IOC AJs: one-way ANOVA *P*<0.0001, Tukey's post hoc: early-mid n.s. *P*=0.97; early-late *P*=0.0001; mid-late *P*=0.0018. *n*=11, 8 and 38 AJs for early, mid and late stages of development, respectively. Data are mean±s.e.m. (C) Each cell-cell boundary included in the vertex model is color coded following the code used in A,B. The bonds representing the cytosolic contractile actomyosin meshworks are represented as dashed magenta lines, with CCs highlighted in blue, PPCs in green and IOCs in gray. The parallel spring schematic represents the tension structure for adhesion and myosin contribution in each cell-cell contact. (D) Heatmap demonstrating the state of intercalation as a function of cytosolic contractile actomyosin meshwork strength (as a fraction of base tension level) (*x*-axis) and the range of contributions from adhesion and myosin intensity measurements (*y*-axis). Spring schematics represent the weight of each adhesion and MyoII in the calculation of tension values for each row. Ommatidia schematics represent the strength of the cytosolic mesh. See Materials and Methods and Table S1 for details. Green represents stable intercalation (E″), red represents failed intercalation (E) and yellow represents a stable four-way junction forming a rosette (E′). Gray points have unstable ommatidia geometry. (E-E″) Simulation snapshots where the tension values cannot drive or stabilize the intercalation (E), where a stable four-way junction is formed (E′) and where a stable intercalation occurs (E″).

Vertex models depend on modeling tension at each AJ, which is a combination of contractility from MyoII and counteracting adhesion forces. Therefore, to set up the vertex model, we quantified the intensity of MyoII at all AJs, and surveyed the principal adhesion molecules that have been shown to be involved in mediating intercellular adhesion in the lens. This includes Ecad and Ncad, as well as Rst and Hbs (Bao and Cagan, 2005; Carthew, 2005; Chan et al., 2017) (Fig. 3A). Intensity values for each of these adhesion molecules were normalized and summed to give a pan-adhesion parameter, relative to the expression levels on the non-shrinking cone cell AJs (Table S1). Given that the expression level of MyoII and adhesion may not directly reflect their contribution to junctional tension (e.g. the existence of MyoII on a junction does not necessarily mean that it is contracting that junction), we used the model to explore the relative weighting (*w*) that MyoII (*w_myo_*) and adhesion (*w_ad_*) contribute to junction tension. We then calculated tension values based on these weighted averages of MyoII and adhesion contributions at the AJs, with MyoII being directly proportional and adhesion inversely proportional to the effective tension of an AJ (see the Computational modeling section in the Materials and Methods for a detailed formula). The adhesion parameter was calculated as the sum of relative intensities of Ecad, Ncad, Hbs and Rst (relative to the non-shrinking cone cell AJs). This approach allowed us to integrate all these adhesion molecules in the model while accounting for the fact that not all AJs in the ommatidium contain Hbs, Rst and/or Ncad.

Where possible, the tension values were estimated experimentally from laser ablation experiments (Fig. 3B). Ecad::GFP, Rst::GFP, Hbs::GFP, Sqh::GFP and Ncad staining intensity measurements were quantified for ommatidia in a post-intercalated state [30% after puparium formation (APF)] (Fig. 3A-A″″, Movies 2 and 3), and normalized to the average of the non-shrinking cone cell AJs. To set up the model, we applied these tension values to simulated ommatidial cell clusters with post-intercalation topologies (Fig. 3C).

We then used the model to explore how cone cell arrangement is affected when varying adhesion and/or contractility through modeling. The tension ranges were obtained by varying the averaging weight by 0, 30, 50, 70 or 100% to reflect the varying ratio of the contribution of adhesion (cyan) and MyoII contractility (magenta) to tension at the AJ (Fig. 3D, *y*-axis). In addition to varying the tension at the AJs, we simulated a range of cytosolic MyoII contractility levels in the primary pigment cells, whereby contractility in these cells applies tensile forces on the cone cell cluster (Blackie et al., 2020) (Fig. 3C, dashed lines; Fig. 3D, *x*-axis). Considering an ommatidium in a stable post-intercalation topology (green zone in Fig. 3D heatmap, Fig. 3E″), a reduction in AJ adhesion (moving down the *y*-axis) causes the topology to switch from post-intercalation topology to the four-way vertex (yellow zone, Fig. 3D,E′) and even back to the pre-intercalation topology (red zone, Fig. 3D,E). By contrast, a reduction in cone cell AJ MyoII contractility (moving up the *y*-axis) coupled with a reduction in primary pigment cell cytoplasmic MyoII (moving left on the *x*-axis) enables the ommatidium to remain in the stable post-intercalation state even for a significant amount of MyoII reduction (diagonal green zone, Fig. 3D). Thus, although a wide range of contractility/adhesion can support stable cone cell intercalation, our model suggests that it can be achieved through adhesion, with minimal input form MyoII. Our model also suggests that stable cone cell intercalation is mechanically influenced by the primary pigment cells (Fig. 3D).

### A limited role for actomyosin contractility during cone cell intercalation

Our vertex model suggests that there are a range of values of MyoII, adhesion and primary pigment cell contractility where intercalation is achieved (green stable zone, Fig. 3D). These range from adhesion dominating with little role for MyoII-dependent contractility (upper left region, Fig. 3D), to MyoII dominating but only with strong contractile forces from the primary pigment cells to stabilize intercalation (lower right region, Fig. 3D). To test which of these is true for the retina, we manipulated MyoII and made predictions from our model for how the tissue would behave for each region. For example, the model predicts that tension in the primary pigment cells could help to stabilize intercalation if MyoII dominates cone cell junction tension; therefore, perturbing MyoII would have a strong effect on intercalation. However, if adhesion dominates cone cell intercalation, perturbing primary pigment cell tension would have only minor effects on cone cell intercalation.

**Fig. 4.**
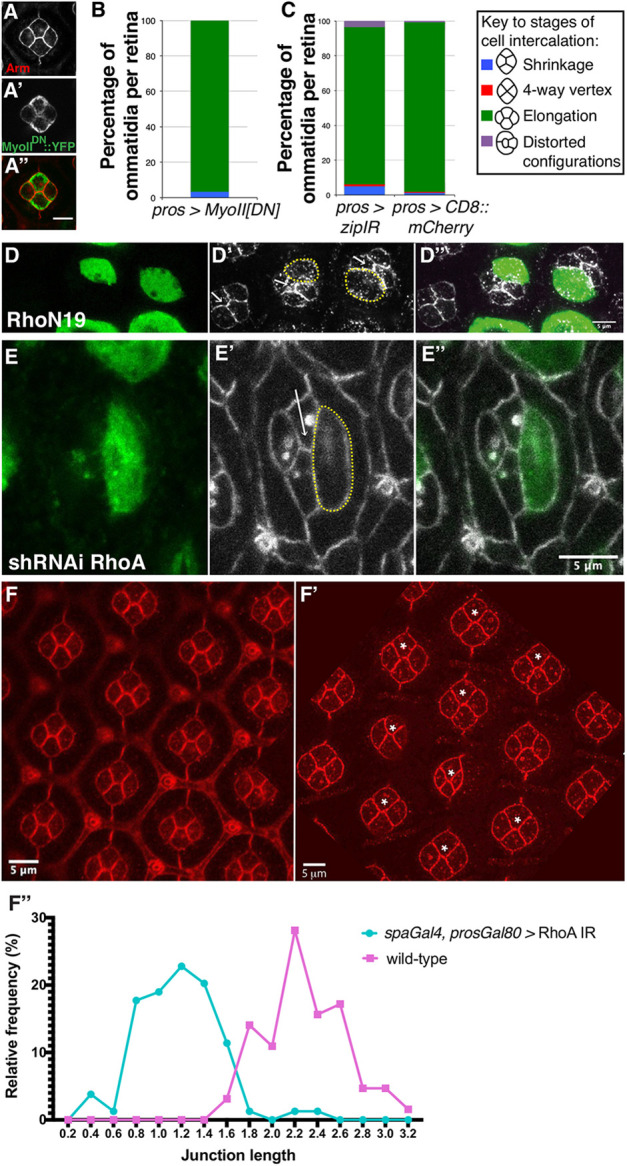
**The RhoA-MyoII pathway is largely dispensable for cone cell intercalation.** (A) *UAS-MyoII^DN^::YFP* expressed under control of *pros-Gal4*. Arm (A), MyoIIDN::YFP (A′) and merged panel (A″). (B) Progression of cone cell intercalation at 29°C when MyoII^DN^ is expressed in cone cells (*n*=4 retinas, 2212 ommatidia). (C) Progression of cone cell intercalation for *UAS-zipIR* expressed under control of *pros-Gal4* alongside matched controls expressing *UAS-CD8::mCherry,* raised at 29°C (*pros-Gal4; zipIR*: *n*=6 retinas; controls: *n*=4 retinas). ‘Other’ category contains any cone cell orientations that do not fit into the other categories (e.g. shift in position of the primary pigment cell junctions relative to the cone cells). (D) GFP-positive cells (circled using a dashed yellow line) express the RhoA^N19^ transgene (green), Ecad::GFP (gray) (D′) and merged panels (D″). White arrows point to the newly extended AJ between Pl and Eq cone cells. (E) GFP-positive cells (circled using a dashed yellow line, *n*=15) express a *RhoA* dsRNAi transgene. White arrows point to the newly extended AJ between the Pl and Eq cone cells. (F) Wild type and (F′) *spaGal4;;prosGal80* genotype expressing *RhoA* dsRNAi in primary pigment cells. Ommatidia marked with an asterisk show a shorter AJ between the Pl and Eq cone cells compared with wild type. The length of these AJs is quantified in F″. For the *spaGal4; prosGal80/RhoA* dsRNAi and *spaGal4; prosGal80/+* genotypes, three retinas each were used for quantification. AJ *n*=80 and control *n*=65. Data are means. Scale bars: 5 μm.

To test these suggestions, we perturbed MyoII expression and activity specifically in the cone cells. A prediction from our model was that a reduction in MyoII levels in the cone cells should not push the cone cells back into a pre-intercalation state (Fig. 3D; a shift up the *y*-axis stays in the ‘green’ post-intercalation zone). Consistent with this, we found that expression of the dominant-negative version of the MyoII heavy chain, Zipper^DN^::YFP (Barros et al., 2003) had a minimal impact on cone cell intercalation, with less than 5% of the ommatidia examined showing a failure to shrink the A/P AJ (Fig. 4A,B). Similarly, expressing double-stranded RNA interference (dsRNAi; IR) against the heavy chain of MyoII in the cone cells interfered with intercalation in less than 10% of cases (Fig. 4C). In these experiments, we were unable to measure how much MyoII activity is inhibited and, therefore, it is possible that we are underestimating the role of MyoII in cone cell intercalation. For this reason and to further probe the contractile actomyosin cytoskeleton, we also examined the requirement for the MyoII activator RhoA. Expressing a dominant-negative RhoA transgene (RhoA^N19^) or dsRNAi targeting this small GTPase in cone cells led to significant relaxation of these cells, which suggests inhibition of the contractile actomyosin cytoskeleton (Fig. 4D,E). However, this did not affect cone cell intercalation. Therefore, taken together, our experiments indicate a minimal requirement for the contractile actomyosin cytoskeleton during cone cell intercalation.

Next, to assess the second prediction of our vertex model, that, if MyoII contractility dominates at the cone cell AJs, then stable intercalation would require pulling tension from the primary pigment cells, we expressed the dsRNAi against RhoA specifically in the primary pigment cells (Fig. 4F). We found that this did not block cone cell intercalation. However, quantification of the length of the AJ that formed between Pl and Eq cone cells after intercalation showed that this AJ was shorter (Fig. 4F′,F″). Specific expression of the dsRNAi transgene was controlled by expressing a UAS-RFP protein (Fig. S2). Thus, although regulation of the contractile actomyosin cytoskeleton in pigment cells promotes lengthening of the AJ between the Eq and Pl cone cells, stable intercalation is still achieved, which, put together with the predictive heatmap generated by our model (Fig. 3D), suggests that this tissue lies towards the upper/left stable region, where intercalation is mainly adhesion dominated.

### Rst and Hbs regulate cone cell intercalation

**Fig. 5.**
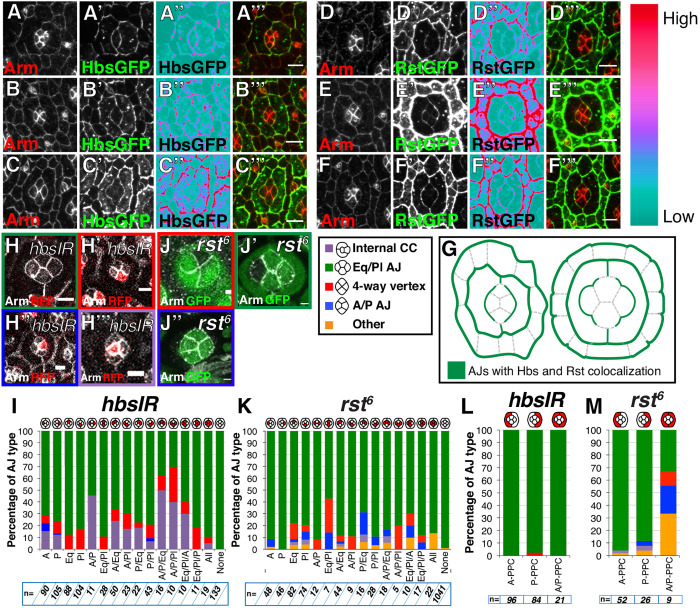
**Rst and Hbs regulate cone cell intercalation.** (A-F‴) Confocal projection through the cone cells (CCs) showing (A-C‴) Hbs::GFP and (D-F‴) Rst::GFP at (A-A‴,D-D‴) AJ shrinking stage, (B-B‴,E-E‴) four-way vertex stage and (C-C‴,F-F‴) AJ elongation stage. In A″,B″,C″,D″,E″,F″ the Ice lookup table was used to visualize variation in levels along the CC-primary pigment cell AJ. A reduction in intensity is seen around the Pl and Eq CCs. A‴,B‴,C‴,D‴,E‴ and F‴ are merged images of Arm (red) and the GFP channel (green). (G) Schematic depicting where Hbs and Rst colocalize. (H) Representative wild-type, control ommatidium from a *hbsIR* mosaic retina. (H′) Eq cell expressing *hbsIR* (red) and stalled at the four-way vertex. (H″) P cone cell expressing *hbsIR* and stalled at the shrinking stage. (H‴) Anterior CC expressing *hbsIR* and showing a cell-sorting phenotype. (I) Quantification of cone cell intercalation in *hbsIR* mosaic ommatidia. dsRNAi-expressing cells are in red. (J) Pl cone cell mutant for *rst^6^* (lacking GFP) stalled at the four-way vertex. (J′) Anterior CC mutant for *rst^6^* (lacking GFP) undergoes normal intercalation. (J″) Primary pigment cells mutant for *rst^6^* (lacking GFP) fail to shrink the A/P CC AJ. (K-M) Quantification of CC intercalation in mosaic ommatidia. dsRNAi for *hbs* and *rst^6^* mutant cells are in red. Scale bars: 5 μm (A-E); 2 μm (H,J).

A process that might induce cone cell intercalation is intercellular adhesion. Among the adhesion molecules that might play a role in these cells are Rst and Hbs. In the case of cone cells, and analgous to how these two factors promote preferential adhesion between interommatidial cells (Bao and Cagan, 2005; Bao et al., 2010), we reasoned that one cone cell expressing Hbs would favor adhesion with one expressing Rst. This type of interaction in *trans* could stabilize the newly created AJ between the Pl and Eq cone cells. To test this hypothesis, we first examined the pattern of Rst and Hbs expression in the cone cells through time. Use of endogenously tagged Rst::GFP and Hbs::GFP showed that both these adhesion molecules are localized at the AJs that the cone cells share with the surrounding primary pigment cells. However, we could not detect these GFP fusions at the AJs between the cone cells (Fig. 5A-F). Enrichment for Rst::GFP and Hbs::GFP was higher at the AJs between primary pigment cells and A/P cone cells than between primary pigment cells and Eq/Pl cone cells (Fig. 5A″-F″, 3A‴,A″″). In addition, both Hbs::GFP and Rst::GFP were enriched at the AJs shared by the primary pigment cells and at the AJs shared between the primary pigment cells and the interommatidial cells, as previously reported (Fig. 5A-F). Although these experiments do not allow us to establish which cells Hbs and Rst are expressed in (i.e. on which side of the AJ), they are compatible with a Rst-Hbs interface taking place between the cone and primary pigment cells (Fig. 5G).

To examine this possibility, we used dsRNAi to decrease the expression of *hbs.* Decreased *hbs* expression in either Eq or Pl cells led to limited defects in intercalation, with a minority (i.e. 10-15%) of clusters failing to elongate the new AJ to complete intercalation (Fig. 5H′,I). Decreasing *hbs* expression in A and P cone cells led to a worse phenotype, where up to 30% of clusters failed to shrink the A/P cone cell AJ (Fig. 5H″,I). In addition, it also led to other defects in cone cell configuration, whereby the mutant cell rounded up to minimize its interface with the flanking primary pigment cell (Fig. 5H‴,I). Taken together, these results confirm those of previous studies (Grillo-Hill and Wolff, 2009). Interestingly, decreasing the expression of *hbs* in all four cone cells had little effect on their intercalation, suggesting that differential Hbs expression among these four cells is required. These results and the pattern of Hbs expression suggest that interactions between the cone and primary pigment cells are involved in cone cell intercalation.

To determine whether Rst is also required in cone cell intercalation, we made use of the *rst^6^* mutant allele. Homozygous *rst^6^* animals are viable and, as noted before (Grillo-Hill and Wolff, 2009), cone cell intercalation occurs normally in these animals. Based on our result that no defects in intercalation are observed when all cone cells are deficient for Hbs, we reasoned that a role for Rst might only be revealed in mosaic situations, when some cone cells are wild type. To test this idea, we recombined the *rst^6^* allele onto an FRT chromosome and generated somatic mutant clones. This approach showed that removing *rst* specifically in the Pl and Eq cone cells led to defects in intercalation (Fig. 5J,J′, quantified in Fig. 5K). In these experiments, up to 40% of the ommatidia presenting both Pl and Eq *rst^6^* mutant cone cells failed to intercalate properly. In addition, a small proportion (less than 10%) of the clusters containing an A or P cone cell mutant for *rst^6^* also showed defects in intercalation. Altogether, the requirement for *hbs* and *rst* found in cone cells, using mosaic analysis, suggests that differential Hbs/Rst activity is necessary for cone cell intercalation.

### Cone cell intercalation is regulated by the primary pigment cells

A and P cone cells deficient for *hbs* expression minimize their AJs with the surrounding pigment cells, suggesting a model whereby adhesion between these cells and the surrounding primary pigment cells is regulated by the Hbs-Rst system. According to this hypothesis, Rst would be required in the primary pigment cells. To test this, we examined ommatidia lacking *rst* in one or both primary pigment cells. First, we confirmed that decreasing *hbs* expression in the primary pigment cells did not affect cone cell intercalation (Grillo-Hill and Wolff, 2009) (Fig. 5L). In contrast, we found that abolishing *rst* expression in both primary pigment cells affected cone cell intercalation in ∼65% of cases (Fig. 5J″,M). In addition, 35% of cases had cone cell configurations resembling those seen when the expression of *hbs* was decreased in the A and P cone cells (Fig. 5H‴). Together with the patterns of Rst::GFP and Hbs::GFP expression (Fig. 5G), these results support the model that the interface between the A and P cone cells and the surrounding pigment cells is promoted by the Hbs-Rst adhesion system, with Hbs in the cone cells engaging in *trans* with Rst expressed in the surrounding primary pigment cells.

### Notch signaling regulates cone cell intercalation

**Fig. 6.**
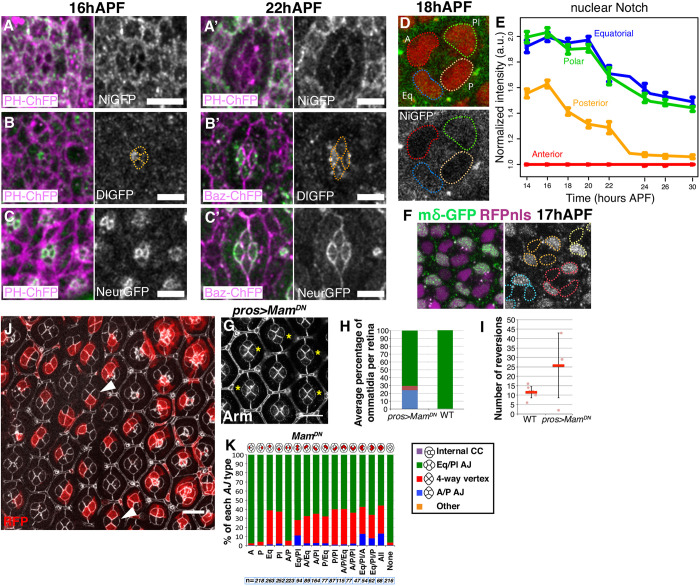
**Notch signaling controls cone cell intercalation.** (A,A′) Timecourse of NiGFP expression (gray) during cone cell intercalation. Cell membranes are labeled with PH::ChFP (purple). (B,B′) Timecourse of Dl::GFP expression (gray) during cone cell intercalation. Cone cells are outlined using a dashed orange line. Cell membranes are labeled with PH::ChFP (purple) in B and with Baz::ChFP in B′. (C,C′) Timecourse of Neur::GFP expression (gray) during cone cell intercalation. Cell membranes are labeled with Baz::ChFP (purple). (D) Representative NiGFP signal (gray) in cone cell nuclei, also labeled using an RFP-nls reporter (red). (E) Quantification of the nuclear signal for Notch in cone cells over time. There is a decrease in the Notch signal in the P cell as intercalation takes place. Data are mean±s.d. (F) Representative staining of the Notch target gene *mδ-GFP* (gray). Cone cell nuclei are labeled using an RFP-nls reporter (purple) and are circled using colored dashed lines with one specific color attributed to each quartet. (G) *UAS-Mam^DN^* expressed under control of *prosGal4*. Yellow asterisks indicate failed intercalation. (H) Progression of cone cell intercalation for retinas expressing *UAS-Mam^DN^* under control of *prosGal4* (*n*=5 retinas, 3433 ommatidia) and for control wild-type flies raised at 25°C (*n*=3 retinas, 1909 ommatidia). (I) Number of reversions between the different stages of cone cell intercalation in wild-type compared with *UAS-Mam^DN^/pros-Gal4* retinas (wild type, *n*=8 ommatidia; *UAS-Mam^DN^/pros-Gal*, *n*=4 ommatidia). Data are mean±s.d. (J) Single cells expressing *UAS-Mam^DN^* marked by presence of RFP (red). Top arrowhead indicates an example of cone cells at a four-way vertex stage when the Eq cell is affected, and the bottom arrowhead indicates a quartet with a A/P AJ when the Eq and Pl cone cells are affected. (K) Quantification of the percentage of ommatidia with each AJ type (A/P, four-way vertex and Eq/Pl) when different combinations of cone cells express *UAS-Mam^DN^. n* numbers are shown for each bar of the graph. Scale bars: 5 μm in A-C′; 10 μm in G,J.

Previous studies have shown that the expression of Hbs in primary pigment cells is regulated by Notch (Bao, 2014). This connection and the finding that Hbs regulates cone cell intercalation prompted us to investigate the role of Notch in this process. First, using live imaging of functional GFP-tagged proteins, we examined the distribution patterns of Notch and its ligand Delta before and after intercalation (Fig. 6A,B) (Corson et al., 2017; Trylinski et al., 2017). At the onset of cone cell intercalation, Notch was present at the apical cortex of all cone cells (Fig. 6A), whereas Dl was highly expressed in the A cell and, to a lesser extent, in the P cell (Fig. 6B), confirming previous observations (Bao, 2014). We also examined the distribution of Neuralized (Neur), a developmentally regulated E3 ubiquitin ligase that is required for Dl endocytosis and Notch receptor activation (Fig. 6C) (Schweisguth, 2004; Weinmaster and Fischer, 2011). Although Neur was detected in all cone cells, we observed a higher level in the A and P cells. These patterns of expression suggest that high levels of Dl in the A cell activate Notch in the P, Pl and Eq cells. To test this suggestion, we monitored Notch signaling over time by measuring either activated nuclear Notch or the Notch intracellular domain (NICD) in the cone cells of living NiGFP pupae (endogenous Notch was tagged with GFP in its intracellular domain so that nuclear GFP reveals NICD; Fig. 6D,E) (Couturier et al., 2012). NICD was detected in the Pl and Eq cells before and after intercalation. In contrast, NICD was detected in the P cell only before intercalation, suggesting that loss of direct contact with the A cell results in loss of Notch receptor activation. Finally, NICD was not detected in the A cell, further suggesting that this cell is the Dl signal-sending cell within the cone cell quartet. This pattern of Notch activity was confirmed by examining the expression of two direct Notch targets, *E(spl)mδ-HLH* (Fig. 6F) and *E(spl)m3-HLH* (not shown), using GFP-tagged proteins. Altogether, our analysis revealed that directional signaling occurs within the cone cell quartet and that a specific change in the pattern of Notch activity correlates with cone cell intercalation.

To test whether Notch signaling contributes to cone cell intercalation, we used a dominant-negative version of Mastermind, Mam^DN^, which blocks transcription downstream of Notch (Giraldez et al., 2002). Expressing Mam^DN^ in all four cone cells led to defects in their intercalation (Fig. 6G,H). Live imaging of these retinas revealed that these defects were due to failures in stabilizing the new Pl/Eq AJ, which led to reversion to the original configuration with an AJ between the A and P cells (Movie 4 and Fig. 6I). In good agreement with our finding that Notch is active in Eq and Pl cells throughout intercalation, examination of ommatidia mosaic for *Mam^DN^* revealed a strong requirement for transcriptional regulation downstream of Notch in each of these two cell types (Fig. 6J,K). From these experiments, we conclude that signaling from either the A or P cell is sufficient to activate Notch in Eq and Pl cells and that this activity is crucial for intercalation. This pattern of Notch signaling and the requirement for Hbs in all cone cells do not suggest a simple Notch-Hbs link in this case. In addition, expressing Hbs in Pl/Eq cone cells also expressing Mam^DN^ did not rescue the intercalation phenotype (not shown).

### Endocytosis is required at all stages of cone cell intercalation

**Fig. 7.**
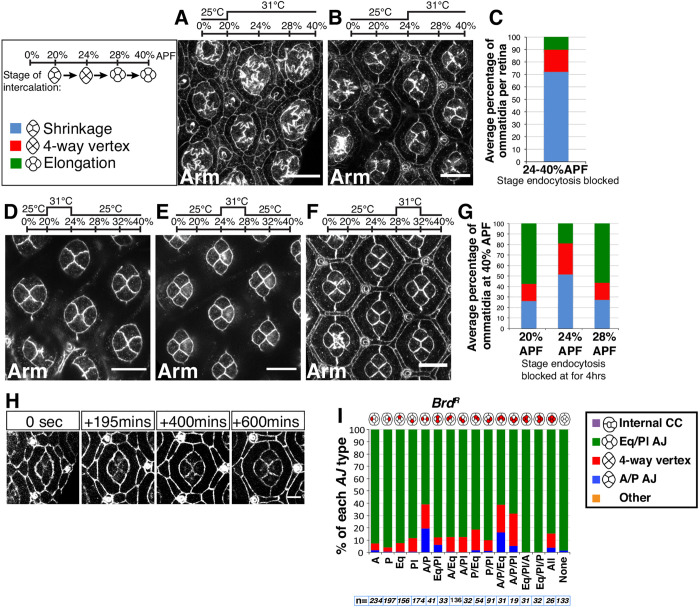
**Endocytosis plays a role in all steps of cone cell intercalation**. (A,B,D-F) Retinas expressing *UAS-shibire^ts^* under the control of *prosGal4* stained for Arm. Flies were raised at 25°C and then transferred to 31°C at (A) 20% APF and (B) 24% APF, and incubated overnight. Flies were transiently transferred to a restrictive temperature for 4 h at (D) 20% APF, (E) 24% APF and (F) 28% APF. (C) Progression of cone cell intercalation in B (*n*=4 retinas, 1442 ommatidia). (G) Progression of cone cell intercalation in D-F (*n*=7, 6 and 6 retinas, respectively; 3352, 3100 and 2797 ommatidia, respectively). (H) Stills taken from a movie of retina expressing *UAS-shibire^ts^* under the control of *prosGal4* with Ecad::GFP to label the AJs*.* (I) Quantification of the percentage of ommatidia with each AJ type (A/P, four-way vertex and Eq/Pl) when different combinations of cone cells express *UAS-Brd^R^*. *n* is indicated for each bar of the graph. Scale bars: 10 μm in A,B,D-F; 5 μm in H.

Our finding suggesting that membrane is recycled from the shrinking junction to the neighboring junctions (Fig. 1H) led us to hypothesize that endocytosis is involved in intercalation by promoting membrane removal during AJ shrinkage. To test this idea, we used the thermo-sensitive Shibire^ts1^ protein (an ortholog of Dynamin). Inhibiting endocytosis in the cone cells as they intercalated caused the AJs they shared to become convoluted (Fig. 7A). Therefore, we conclude that endocytosis regulates membrane turnover during cone cell intercalation. Additionally, cone cell intercalation was blocked either during AJ shrinkage (72% of cases) or at the four-way vertex (18% of cases) (Fig. 7B,C) when assayed at a stage where, in wild-type retinas, all the ommatidia should have completed intercalation.

In order to refine this analysis, we then inhibited endocytosis for only 4 h during each of the three stages of cone cell intercalation: AJ shrinkage, four-way vertex and AJ elongation (Fig. 7D-F). In these experiments, intercalation either stalled or reverted to an earlier stage in a significant number of cases (Fig. 7G). Time-lapse imaging confirmed that inhibition of endocytosis caused a failure in cone cell A/P AJ shrinkage (Movie 5). In agreement with our quantifications (Fig. 7G), live imaging revealed that inhibiting endocytosis at the four-way vertex stage (24% APF) led to reversions to the initial cone cell configuration (Fig. 7H). Taken together, these results demonstrate that endocytosis is required for stable intercalation to proceed between cone cells. AJ convolution is compatible with an excess of plasma membrane, which is consistent with a role for endocytosis in shedding membrane during slow intercalation.

Another aspect of cone cell intercalation that is expected to be affected when blocking endocytosis is the Notch pathway, because Notch signaling requires Dl endocytosis. To test this, we specifically targeted Dl endocytosis by overexpressing a stabilized version of Bearded (Brd) (Leviten et al., 1997), called Brd^R^ (Perez-Mockus et al., 2017). We found that simultaneous expression of Brd^R^ in A and P cone cells led to defects in intercalation in 40% of cases, whereas expressing Brd^R^ in either the A or P cell rarely led to defective intercalation (Fig. 7I). Thus, next to promoting membrane removal, endocytosis is also required for Notch activity in cone cell intercalation.

## DISCUSSION

Most instances of epithelial cell intercalation studied so far have focused on rapid neighbor exchanges, occurring over periods of tens of minutes, as is the case in the fly germband (Bertet et al., 2004; Zallen and Wieschaus, 2004), or over a couple of hours, as during kidney tubule morphogenesis and early retinal development (Lienkamp et al., 2012; Robertson et al., 2012). Intercalation of cone cells in the fly is much slower, because it unfolds over ∼10 h. Combining mathematical modeling and genetics experiments, we found that slow intercalation involves regulations that appear more complex than those found to control faster intercalation (Heisenberg and Bellaiche, 2013; LeGoff and Lecuit, 2015). Our results show that cone cell intercalation requires transcription downstream of Notch in two of the four intercalating cells. We also found that cone cell intercalation shows a low requirement for the contractile MyoII pathways, relying more instead on adhesion, regulated by Rst and Hbs. In addition, our experiments suggest that membrane endocytosis is essential for stable intercalation by shedding plasma membrane during AJ shrinkage and by promoting Notch activation. Finally, we present evidence that cone cell intercalation involves processes extrinsic to the cone cell quartet, involving tensile force and adhesion in neighboring cells.

### The RhoA-MyoII axis is largely dispensable for cone cell intercalation

We found a small enrichment for MyoII at the shrinking cone cell AJ, suggesting that it contributes to inducing shrinkage of the AJ over time. In addition, inhibiting the activity and expression of this motor protein led to cone cell intercalation defects in ∼10% of the ommatidia we examined. Therefore, MyoII plays a limited role during cone cell intercalation. However, we cannot exclude the possibility that residual MyoII levels are present in our genetic perturbations that are sufficient to promote cone cell intercalation. Our perturbations of RhoA, an upstream regulator of the actomyosin cytoskeleton, show that, although RhoA inhibition in cone cells leads to relaxation of their apical profile, intercalation is not impacted. These experiments also support our model in which MyoII is minimally required in cone cell intercalation. This conclusion is also consistent with work revealing that normal cone cell intercalation requires Ncad to downregulate MyoII at the AJs shared between cone cells (Chan et al., 2017). Therefore, irrespective of potential redundancies in MyoII activation in retinal cells, this work and our present study suggest that MyoII concentration/activity needs to be limited at cone cell AJs for intercalation to proceed normally. This situation is somewhat analogous to that reported in the fly notum, where lowering MyoII activity allows for tissue fluidity (i.e. neighbor exchange) (Curran et al., 2017). However, unlike in the notum, AJ remodeling in cone cells is not stochastic but deterministic.

### Adhesion regulates cone cell intercalation

We found that both Hbs and Rst regulate cone cell intercalation. However, the general adhesion logic at play between cone cells, and also between them and the surrounding primary pigment cells is not clear. Hbs is required in all individual cone cells, and our work argues that it plays a role in stabilizing the new AJs created between Eq and Pl cells after intercalation. Rst in Eq/Pl cells also contributes to regulating the creation of these AJs. Therefore, it is possible that a Hbs/Rst interaction takes place in *trans* between the Eq/Pl cells to stabilize the newly created AJs. However, Rst::GFP and Hbs::GFP showed no detectable enrichment at these AJs. In fact, one of the strongest effects on cone cell intercalation was seen when *rst* was removed from the primary pigment cells. Rst::GFP and Hbs::GFP were detected at the AJs between these cells and cone cells. Therefore, we hypothesize that Hbs-Rst interactions take place between these two cell types. In this hypothesis, we envisage that Hbs expressed in cone cells contacts Rst expressed in primary pigment cells. Primary pigment cells also express Hbs, which accumulates at the AJs that they share with interommatidial cells (Bao and Cagan, 2005). Therefore, our work raises the possibility that primary pigment cells express both Rst and Hbs, and that these proteins have a planar polarized distribution in this cell type. This possibility is consistent with our finding that both Rst and Hbs are strongly localized to AJs between two primary pigment cells.

### Notch signaling between cone cells regulates intercalation

In addition to a role for Hbs and Rst, our work also revealed that a Notch-Dl code exists between the four cone cells that regulates their intercalation. In this code, Dl signals from A or P cells, whereas Notch is activated and required in Eq and Pl cells. This function for Notch requires transcription because it is blocked by Mam^DN^. Previous work established that Notch can induce Hbs expression (Bao, 2014). However, the pattern of Notch activation in Eq/Pl cells and the requirement for Hbs in all cone cells does not suggest a Notch-Hbs pathway in this case. Thus, more work is required to understand how Notch regulates intercalation and how expression of Hbs and Rst is controlled in cone cells. We also note that Notch activation has previously been placed downstream of the Hbs-Rst system in the eye (Singh and Mlodzik, 2012). Such regulation could also be at play during cone cell intercalation. Considering Notch signaling between cone cells, it is possible that perturbing Hbs/Rst expression in these cells might affect the Notch-Dl interface by changing the surface contact that these cells share.

### Endocytosis and cone cell intercalation

Our work reveals a key function for membrane endocytosis during cone cell intercalation. This could be due to several reasons. Blocking endocytosis in primary pigment cells leads to defects in Rst localization (Johnson et al., 2008) and, by analogy, blocking endocytosis in cone cells could also affect the Rst/Hbs pathway and, thus, intercalation. Similarly, endocytosis of Dl is required to activate the Notch pathway (Schweisguth, 2004; Weinmaster and Fischer, 2011). Indeed, blocking Dl endocytosis by expressing Brd^DN^ in A/P cone cells interferes with intercalation. Endocytosis is also likely to be required to control the turnover of Ecad/Ncad in cone cells and promote plasma membrane removal during AJ shrinkage. For example, as these cells intercalate, Ecad becomes depleted from the AJs between cone cells and Ecad overexpression in Eq/Pl cone cells blocks intercalation (Carthew, 2005). It is possible that endocytosis promotes this inhibition. Consistent with this type of model, similar regulation of Ecad endocytosis takes place in the fly embryo during intercalation to shrink AJs (Levayer et al., 2011).

### Extrinsic regulation of cone cell intercalation

In addition to showing a dominant role for adhesion over MyoII in regulating cone cell intercalation, our vertex model also predicts that external tensile forces in the surrounding primary pigment cells could play a role (Fig. 3D, moving left on the *x*-axis in the heat map). The primary pigment cells present medial meshworks of actomyosin that are contractile (Blackie et al., 2020). This contractility regulates the width of the apical profiles of these cells and would be expected to pull onto the AJs that these cells share with A and P cone cells. Perturbing the actomyosin cytoskeleton in primary pigment cells by inhibiting *RhoA* expression led to a decrease in the length of the cone cell central contact but did not completely block intercalation (Fig. 4E,F). Taken together with the fact that reduction of MyoII in the cone cells does not inhibit intercalation but reduction of adhesion does, this suggests that ommatidia lie in the upper/left section of the predictive heatmap generated by our model, whereby this slow mode of intercalation is driven dominantly by adhesion within cone cells.

## MATERIALS AND METHODS

### Fly strains

Flies were raised on standard food at 18°C. Crosses were performed at 25°C or 29°C as stated. The following fly strains were used:

*;Ecad::GFP* (Huang et al., 2009)

*sqh^AX3^; sqh>sqh::GFP* [BL 57144 (Royou et al., 2002)]

*;Sp/CyO;pros-Gal4/TM6* (a gift from Tiffany Cook, Wayne State University School of Medicine, Detroit, MI, USA)

*;GMR-Gal4* (Freeman, 1996)

*;;UAS-shibire^ts1^* [BL 44222 (Koenig and Ikeda, 1983)]

*;UAS-YFP-MyoII^DN^* (Barros et al., 2003)

*rst^6^* (Wolff and Ready, 1991)

*ubiGFP,FRT19A;eyFlp*

;*eyFlp;FRT40,GMR-myrRFP/CyO*

*rst::GFP* (BL 59410)

*;hbs::GFP* (BL 65321)

*;UAS-hbsRNAi* (VDRC 105913, VDRC 40898)

*;UAS-rstRNAi* (VDRC 951, VDRC 27223)

*hsflp;;act>CD2>GAL4,UAS-RFP* (BL 30558)

*hs-flp;actin>y>gal4,UAS-mCherry;armGFP/TM6*

*;;UAS-Mam^DN^* (BL 26672; Helms et al., 1999)

*Neur::GFP*, a BAC transgenic line with two copies of GFP-tagged Neur (Perez-Mockus et al., 2017)

*ubi-Baz::mCherry* (Bosveld et al., 2012)

*UAS-Brd^R^* (Perez-Mockus et al., 2017)

*Dl::GFP*, a GFP knock-in allele (Corson et al., 2017)

*Ni::GFP*, a GFP knock-in allele (Trylinski et al., 2017)

*E(spl)m3-HLH::GFP*, a GFP knock-in line (Couturier et al., 2019)

*E(spl)mδ-HLH::GFP,* a BAC transgenic line expressing GFP-tagged E(spl)m *δ* -HLH (Couturier et al., 2019)

*w;;FRT82B, ubi-nlsRFP* (BL-30555)

*;;UAS-dsRNAi RhoA* (BL-27727)

*;UAS-dsRNAi zip* (VDRC 7819)

*spaGal4* (BL-26656)

*;;prosGal80* (this study)

*w;UAS-RhoA*^N19^ (BL-7328).

### Transgenes

The *prospero* eye enhancer (Charlton-Perkins et al., 2017) was PCR-amplified from the *prosGal4* plasmid (a gift from Tiffany Cook) and CACC was added to the 5′ end using the following primers: 5′ CACCATCTGTGACGAAGACACTCGTTTTGAG 3′ and 5′ TCGATTGCCAGGAAGTGCAGG 3′. The PCR fragment was cloned into the *pENTR™/D-TOPO* vector (Invitrogen K240020) and then sequence verified. The Gateway Cloning System (Invitrogen) was used to insert the *pros* enhancer into the *pBPGal80Uw-6* destination vector (Addgene plasmid 26236) to generate a *prosGal80* plasmid. Transgenic flies were generated using standard procedures (Bestgene) and *prosGal80* was inserted into attP2.

### Clonal analysis

To generate mosaic ommatidia, *hs-flp;;actin>CD2>gal4,UAS-RFP* or *hs-flp;actin>y+>gal4,UAS-mCherry;arm-GFP/TM6* was crossed to *UAS* transgenes of interest. Flies were heat shocked at the third-instar larval stage at 37°C for 10-15 mins and then dissected 2-3 days later at 40% APF (25°C). To generate *rst^6^* mosaics, *rst^6^* was recombined onto an FRT19A chromosome, which was then used in combination with an FRT19A, ubi-GFP; eyFLP strain. The Coin-FLP system (Bosch et al., 2015) was used to generate mosaics expressing dsRNAi targeting RhoA. Animals were raised at 18°C.

### Inhibition of endocytosis using *shibire^ts1^*

*UAS-shibire^ts1^* flies were crossed to *prosGal4* flies and raised at 25°C until the stated time of development, then transferred to 31°C to block endocytosis for either 4 h or overnight. Retinas were dissected at 40% APF and scored for progression of cone cell intercalation.

### Antibodies and immunostaining

Pupae were staged at 25°C or 29°C to 40% APF; retinas were then dissected in PBS on ice and fixed in 4% paraformaldehyde for 20 min at room temperature (RT). Retinas were washed in PBS-Triton 0.3% (PBS-T) and then stained with primary antibody in PBS-T for 2 h at RT or overnight at 4°C. Retinas were washed in PBS-T and then stained with secondary antibodies for 2 h at RT. Retinas were then mounted in Vectashield (Vectorlabs).

The following antibodies were used: mouse N2 A71 anti-Armadillo (1:50), deposited in the Developmental Studies Hybridoma Bank (DSHB) by E. Wieschaus (DSHB Hybridoma Product N2 7A1 Armadillo) (Peifer and Wieschaus, 1990); DCAD2 anti-E-cadherin (1:50), deposited in the DSHB by T. Uemura (DSHB Hybridoma Product DCAD2) (Yoshida-Noro et al., 1984); and DN-Ex anti-N-cadherin (1:50), deposited in the DSHB by T. Uemura (DSHB Hybridoma Product DN-Ex) (Iwai et al., 1997); combined with mouse or rat secondary antibodies conjugated to Dy405 (715-475-151), Alexa488 (715-545-151, 712-545-153), Cy3 (715-165-151, 712-165-153) or Alexa647 (715-605-151, 712-605-153) (Jackson ImmunoResearch) as appropriate, used at 1:200.

Images of fixed retinae were acquired on a Leica SPE, Leica SP5 or Leica TCS SP8 confocal microscope. A 40× oil objective was used for imaging of whole retinas for quantification and a 63× oil objective was used for higher-magnification images.

### Image processing

All images presented were processed using FIJI (Schindelin et al., 2012) and Adobe Photoshop CS4. Graphs were produced in Excel (Microsoft), GraphPad Prism 7 (GraphPad) or MATLAB R2017a (Mathworks). Figures were mounted in Adobe Illustrator CS4.

### Statistical tests

Statistical tests were performed in GraphPad Prism 7. Data were compared using a Student's *t*-test (paired or unpaired as appropriate) or one-way ANOVA with Tukey's post-hoc tests. Differences were statistically significant at *P*<0.05.

### Time-lapse imaging

*Ecad::GFP*, *Ecad::GFP/Ecad::GFP;UAS-Mam^DN^/pros-Gal4* or *Ecad::GFP/+;UAS-shibire^ts1^/pros-Gal4* flies were staged to between 10% and 20% APF at 25°C and the pupal case was removed at the dorsal end to expose the retina. Pupae were mounted on Blu Tack with the retina facing upwards and covered with a coverslip, as previously described (Fichelson et al., 2012; Couturier et al., 2014). Time-lapse imaging was performed on a Zeiss inverted microscope with an Andor spinning disc using a Plan Neofluar 100×/1.3 Ph3 oil immersion objective. Images were acquired using ImageJ Micromanager software (Edelstein et al., 2010).

Retinas were imaged for a minimum of 12 h taking a *z-*series in 1 μm sections every 5 mins. Drift in *xy* and *z* was corrected manually. Images were post processed in FIJI to further correct for drift. For *Ecad::GFP;UAS-shibire^ts1^/pros-Gal4*, flies were raised at 25°C until 15-20% APF and then transferred to the microscope and incubated at 31°C to stimulate endocytosis inhibition as soon as imaging began. Nuclear Notch levels were measured over time in *NiGFP* pupae using small *z*-stacks centered at the nucleus level. Image acquisitions were performed at 20±2°C, using a laser-scanning confocal microscope (LSM780; Zeiss) with a 63× (Plan APO, N.A. 1.4 DIC M27) objective.

### Laser ablation

*Ecad::GFP* pupae were raised and mounted as for the time-lapse imaging experiments described earlier. Ablations of AJs between primary pigment cells and between interommatidial cells were performed using a Zeiss LSM880 microscope with a Plan Apochromat 63×/NA1.4 oil objective using 740 nm multiphoton excitation from a Ti-sapphire laser. A region of interest (ROI) of 3×3 pixels was drawn over an AJ and ablated with 5-10% laser power at the slowest scan speed for one iteration. Images were acquired every 1 s after ablation. Settings were optimized by imaging *sqh^AX3^;sqh::GFP* flies during ablation to ensure that only the AJ-associated MyoII was removed and that the medial meshwork of MyoII remained intact. The AJs repaired after every instance of ablation, indicating that the cell was not damaged. Positions of the two adjoining vertices after ablation were manually tracked using FIJI and the distance between them calculated at each frame after ablation. Recoil velocity was calculated by a linear fit across the first frames after ablation. One-way ANOVA was performed in GraphPad Prism7 to compare stages.

### Cone cell area through time

The Tissue Analyser FIJI plug-in (Aigouy et al., 2010) with manual correction was used to segment the cone cells on a time-lapse (5 min/frame) of *;Ecad::GFP* retinas. The areas of individual cone cells were then measured and averaged across four ommatidia.

### Length of cone cell axes over time

The perimeter of the cone cell cluster was traced manually using the Freehand selection tool in FIJI on every 20th frame (100 mins) of time-lapse of *;Ecad::GFP* retinas. To measure the lengths of the A/P and Eq/Pl axes, an ellipse was fitted over the cone cells, measured over time and then expressed as a ratio. Measurements were averaged for each time point over 13 time-registered ommatidia from two independent retinas.

### AJ perimeter measurements

AJ lengths were taken as the intervertex distance and were measured manually on each frame of time-lapse movies of *;Ecad::GFP* retinas using the line tool in FIJI. Measurements were averaged from 13 time-registered ommatidia from two independent retinae.

### Cross-correlation of AJ length

Curves of AJ length over time for cone cells were smoothed by taking a five-point moving average. Data were detrended by taking the running difference to find the change in AJ length over time. Cross-correlation was performed using the R statistical package with the ccf function. The mean cross-correlation function was calculated as the average correlation coefficient at each time lag across 13 ommatidia from two independent retinas.

### Intensity at the AJs

*sqh^AX3^;sqh-sqh::GFP;*/*rst::GFP;;/;hbs::GFP;*/*EcadGFP* or *CantonS* flies stained for Ncad were staged to 30% APF and retinas were dissected, fixed and stained for Arm. Retinas were imaged on a confocal microscope (Leica SP5) using the same settings for each retina. A *z*-projection was generated over the depth of the AJs in FIJI. A seven-pixel-wide line was drawn over each AJ in the Arm channel and the intensity of each channel was averaged over this line, except for the cone-primary pigment cell AJs: because Sqh::GFP was present only on one side of the AJ, a four-pixel (approximately half of seven) line was drawn over the Sqh::GFP channel. To control for variations in staining and/or imaging, all individual values within each image were normalized by dividing by the average value for the adjoining cone cell-cone cell AJs for that image. One-way ANOVA followed by Tukey's post-hoc tests for pairwise comparison were performed using GraphPad Prism 7. For comparison of cone cell-cone cell AJs, a paired Student's *t*-test was performed paired by ommatidia.

### Scoring cone cell intercalation

Retinas were dissected at 40% APF, fixed and stained for Arm to label the AJs. Whole retinas were imaged with a 40× objective taking a *z*-series in 0.5 μm slices in two tiles. Tiles were manually aligned and combined using the Align3 TP plugin in FIJI. Ommatidia were manually scored for stage of cone cell intercalation across each retina. Stage was determined by comparing the position of the central cone cell-cone cell AJ and the two AJs shared by the primary pigment cells (parallel=AJ shrinkage stage; perpendicular=*AJ* elongation stage). Percentages were calculated for each retina and the average percentages and standard deviations were then calculated for each genotype. For large clones, ommatidia were compared from inside and outside the clones. For single-cell clones, the relative position of cells (A, P, Eq and Pl) was recorded and ommatidia were grouped into categories based on which cells were affected.

### Scoring switches between stages of cone cell intercalation

For the *Ecad::GFP* and *;Ecad::GFP/Ecad::GFP;UAS-Mam^DN^/pros-Gal4* time-lapse movies, ommatidia were scored for which stage of cone cell intercalation they were in for each frame of the movie (5 min intervals). A ‘switch’ in a given frame was defined as being scored at a different stage to that of the previous frame. The total number of switches was measured from 10% of retinal development (before cone cell intercalation) for ∼12 h (until after cone cell intercalation is completed in the wild type).

### Particle image velocimetry

*sqh^AX3^;sqh-sqh::GFP* ommatidia were imaged on a Zeiss LSM880 microscope with a Plan Apochromat 63×/NA1.4 oil objective using Airyscan detectors to increase resolution. Movies were processed by bleach correction and Gaussian blur, and registered with the Stack-reg plug-in (Thevenaz et al., 1998) if needed in FIJI. Particle image velocimetry (PIV) analysis was performed using the FIJI PIV plug-in (Tseng et al., 2012), by choosing an 8×8 pixel window with a time lag of 4.34 s. Cell contours were tracked either by using the Tissue Analyzer plug-in (Aigouy and Le Bivic, 2016) in FIJI or manually in FIJI to segment the cone cells. The angles of each PIV vector within the cone cells over time were plotted in a polar histogram in MATLAB to show the lack of overall directional flow.

### Computational modeling

The ommatidium was modeled using the well-established cellular vertex model (Farhadifar et al., 2007). In vertex models, cells are described by the polygons formed by their contacting edges. These edges represent the attached contacting surfaces (membrane and actomyosin cortices) of both cells. The positions of vertices forming all edges are traced, and the energy minimization is carried out over these positions. The energy of the system is defined by the combined energy contributions of: (1) cell area conservation, i.e. the deviation of each cell from its ideal size; and (2) the combination of tension and adhesion energies at each contacting cell-cell boundary (Eqn 1):(1)
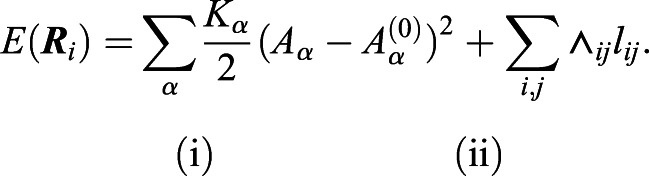


Here, *E*(***R***_*i*_) is the total energy of the system for a given set of vertex positions (***R***_*i*_) that the algorithm minimizes. The system is composed of *N_c_* cells (a=1…*N_c_*) and *N_v_* vertices (*i*=1…*N_v_*). For area conservation term (i), *K*_α_ is the elasticity coefficient, A_α_ is the current area of the cell and A^(0)^_α_ is the ideal area. For the tensile/adhesive contact energy contribution (ii), Λ*_ij_* is the line tension coefficient for the junction couple (*i*,*j*) and *l_i__j_* is the length of the junction between vertices. For the simulations in this article, the base tension is Λ^0^_*ij*_=0.26, base A^(0)^_α_ is 1.0 (see Table S1 and Fig. 3) and *K_α_* is 1.0. The small cone cell area was set to A^(0)^_α_ and remaining cell sizes were scaled accordingly with experimental size scaling measurements (Table S1). The symmetrical side junctions in between the cone cells were selected as the base, similar to experimental intensity measurements depicted in Fig. 3A-A″″. A weighted average of the adhesion and myosin intensity measurements (Fig. 3B′) was used as a surrogate for scaling tension values (Table S1). To scale the tension contribution of adhesion to a cell-cell junction, the base tension level was scaled inversely to the normalized adhesion intensity of the junction. For scaling MyoII levels, the tension was scaled directly proportional to normalized MyoII intensity, as given in Eqns 2-4:(2)
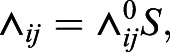
(3)
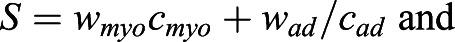
(4)
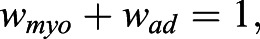
where *S* is a scaling factor for the line tension, (*w_xx_*) are the averaging weights of myosin (*myo*) and adhesion (*ad*) and (*c_xx_*) are the normalized intensities. During the post-intercalation (late) phase, the primary pigment cell contact and contacts between interommatidial cells on the sides of the ommatidium were not significantly different, whereas the tension on the top and bottom interommatidial cell contacts was 51% higher (Fig. 3C). The laser ablations depict the true tension of a bond, and model parameters were scaled accordingly where information was available (Table S1). Simulations were carried out on a setup of 91 connected ommatidia with fixed boundaries, and analysis was performed on the central ommatidium.

## Supplementary Material

Supplementary information

Reviewer comments
