# Supplementary material for: A combination of Notch signaling, preferential adhesion and endocytosis induces a slow mode of cell intercalation in the Drosophila retina
Source: Development. 2021 May 17;148(10):dev197301. doi: 10.1242/dev.197301 (PMC8180261; doi:10.1242/dev.197301)
Supplement: Supplementary information [file develop-148-197301-s1.pdf]

Table S1

Cell type	Ideal area as a fraction of $A^{(0)}_{\alpha}$			
	**Post-intercalation**			
Pigment cell (PPC)	8.7			
Cone cell (CC) sides	1			
Cone cell (CC) top/bottom	1.3			
(IOCs)	1			
**Contact type**	**Post- intercalation**			
	c_{ad}	c_{myo}	S if $w_{ad} = 1$ $w_{myo} = 0$	S if $w_{ad} = 0$ $w_{myo} = 1$
CC-CC side contact	1.00	1.00	1.00	1.00
CC-CC central contact	1.08	1.06	0.93	1.06
CC – PPC (Eq/Pl)	1.79	1.52	0.56	1.52
CC – PPC (A/P)	2.11	1.52	0.47	1.52
PPC-PPC contact	3.25	1.33	0.31	1.33
PPC - IOC contact	4.83	1.93	0.21	1.93
IOC-IOC (A/P)	2.37	2.24	0.19*	1.33*
IOC-IOC (Eq/Pl)	2.24	2.30	0.29*	2.02*
all else	1.00	1.00	1.00	1.00

Development • Supplementary information

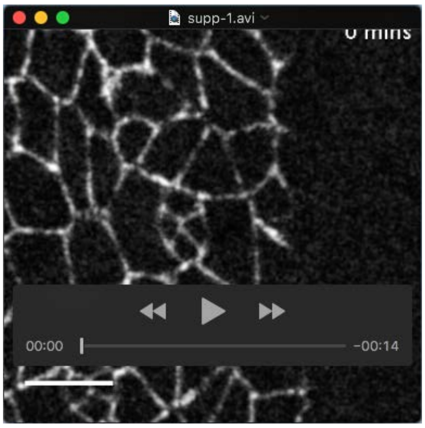

Movie 1: Time-lapse of a representative cone cell intercalation. Cells are labeled using Ecad::GFP. Frame interval = 5 minutes. Scale bar = 5µm.

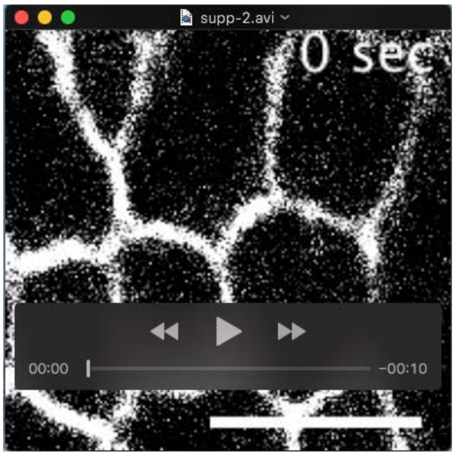

Movie 2: Representative laser ablation of an inter-Interommatidial cell AJ. Cells are labeled using Ecad::GFP. Frame interval = 1 second. Scale bar = 5µm.

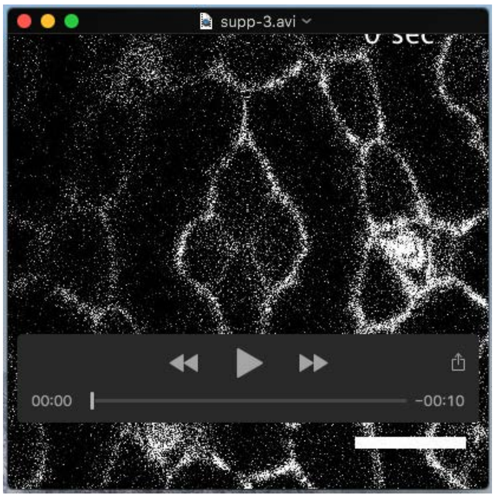

Movie 3: Representative laser ablation of an Inter-primary pigment cell AJ. Cells are labeled using Ecad::GFP. Frame interval = 1 second. Scale bar = 5µm.

Development • Supplementary information

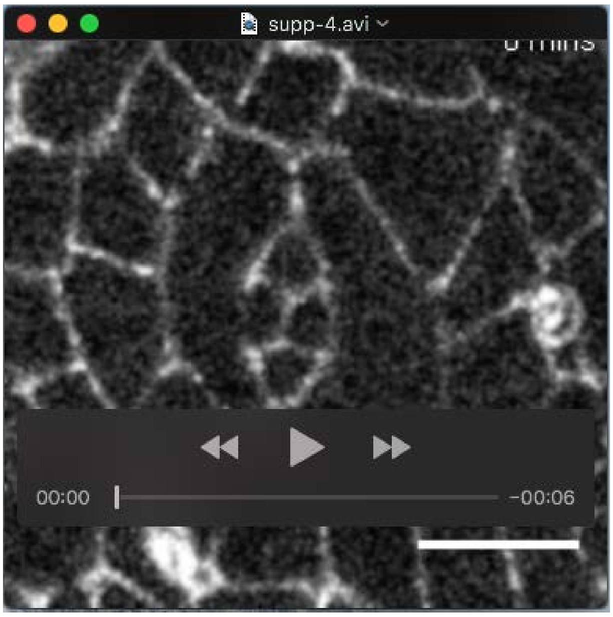

Movie 4: Representative cone cell quartet expressing Mam^{DN}. Cells are labeled using Ecad::GFP. Frame interval = 5 minutes. Scale bar = 5μm.

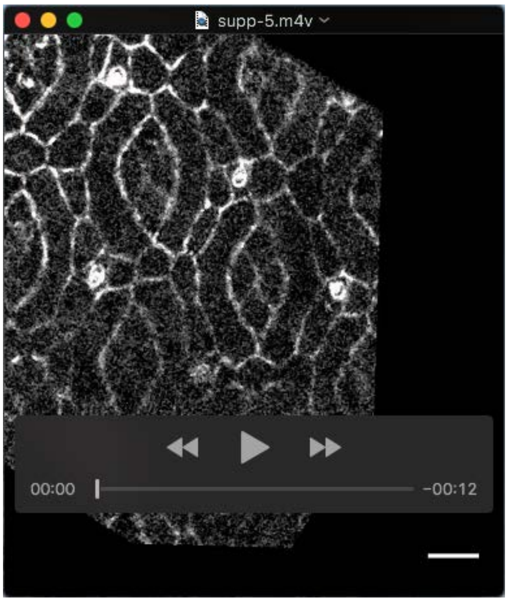

Movie 5: Time-lapse of a representative cone cell intercalation upon endocytosis inhibition. Cells are labeled using Ecad::GFP. Movie starts at a timepoint when in WT the majority of cone cells would be at the four-way vertex and would expand their Eq/Pl junctions. Note how when endocytosis is blocked, intercalation is stalled or cone cells even revert their contacts. Frame interval = 10 min. Scale bar = 5μm.

Development • Supplementary information

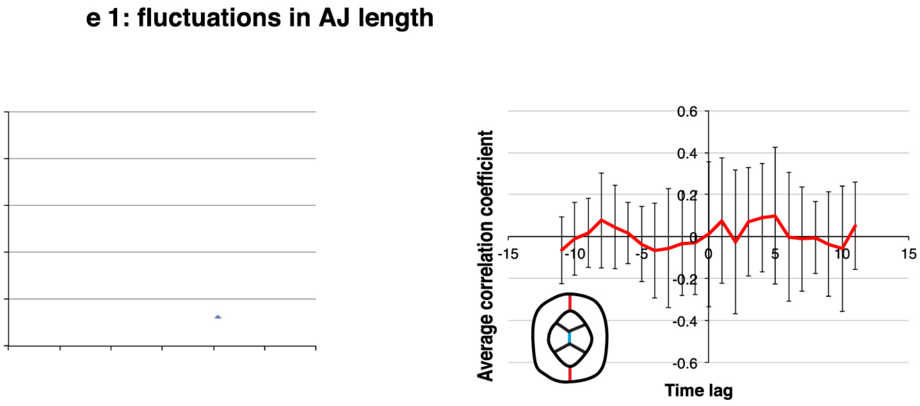

Figure S1: Fluctuations in AJ length.

(A) Length of central cone cell AJ and the primary pigment cell AJs of one ommatidium showing negative correlation. Pearson's correlation coefficient for this example: r=-0.74 for PI primary-primary cell and r=-0.67 for Eq primary-primary pigment cell. Average correlation coefficient: r=-0.6±0.19 (mean±S.D.) (n=13 ommatidia). **(B)** Average cross-correlation of rate of change in the length of the central cone-cone AJ (shown in blue in schematic) with the primary-primary AJs (shown in red in schematic) (n=13 ommatidia). Error bars = S.D.

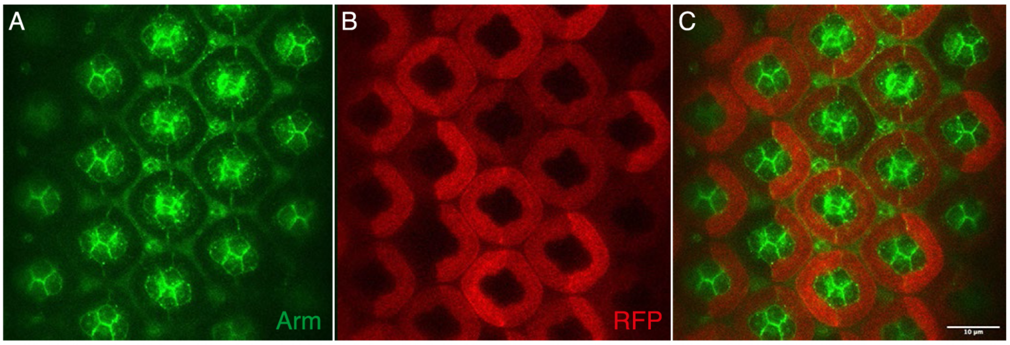

Figure S2: Specific transgene expression in the primary pigment cells. The *spaGal4, prosGal80* strain allows specific expression of a *UAS-RFP* transgene in the primary pigment cells.
