# Supplementary material for: A combination of Notch signaling, preferential adhesion and endocytosis induces a slow mode of cell intercalation in the Drosophila retina
Source: Development. 2021 May 17;148(10):dev197301. doi: 10.1242/dev.197301 (PMC8180261; doi:10.1242/dev.197301)
Supplement: Reviewer comments [file develop-148-197301_review_history.pdf]

A combination of Notch signaling, preferential adhesion and endocytosis induces a slow mode of cell intercalation in the *Drosophila* retina.

Laura Blackie, Melda Tozluoglu, Mateusz Trylinski, Rhian F. Walther, François Schweisguth, Yanlan Mao and Franck Pichaud
DOI: 10.1242/dev.197301

Editor: Thomas Lecuit

Review timeline
Original submission: 24 September 2020
Editorial decision: 27 October 2020
First revision received: 3 March 2021
Accepted: 9 April 2021

Original submission

First decision letter

MS ID#: DEVELOP/2020/197301

MS TITLE: A combination of Notch signaling, preferential adhesion and endocytosis induces a slow mode of cell intercalation in the Drosophila retina.

AUTHORS: Laura Blackie, Melda Tozluoglu, Mateusz Trylinski, Rhian Faye Walther, Yanlan Mao, François Schweisguth, and Franck Pichaud

I have now received all the referees reports on the above manuscript, and have reached a decision. The referees' comments are appended below, or you can access them online: please go to BenchPress and click on the 'Manuscripts with Decisions' queue in the Author Area.

The overall evaluation is overal quite positive and we would like to publish a revised manuscript in Development, provided that the referees' comments can be satisfactorily addressed. I draw attention to the model that 2 of the referees feel is not compatible with current data and not well suited. Please attend to all of the reviewers' comments in your revised manuscript and detail them in your point-by-point response. If you do not agree with any of their criticisms or suggestions explain clearly why this is so.

We are aware that you may currently be unable to access the lab to undertake experimental revisions. If it would be helpful, we encourage you to contact us to discuss your revision in greater detail. Please send us a point-by-point response indicating where you are able to address concerns raised (either experimentally or by changes to the text) and where you will not be able to do so within the normal timeframe of a revision. We will then provide further guidance. Please also note that we are happy to extend revision timeframes as necessary.

© 2021. Published by The Company of Biologists under the terms of the Creative Commons Attribution License (https://creativecommons.org/licenses/by/4.0/).

Reviewer 1

Advance summary and potential significance to field

In this paper, Blackie et al. identify several mechanisms that contribute to junctional remodeling during neighbor exchanges between the four cone cells in the Drosophila retina, a complex epithelium composed of multiple distinct cell types. The authors show that myosin is enriched at the shrinking junction between the polar and equatorial cone cells, but perturbing myosin function has only a minor effect on cell intercalation. Shrinkage of this junction correlates with expansion of the adjoining junctions and expansion of the 4-cell cluster along the same dimension. They show that blocking endocytosis prevents normal junction shrinkage and intercalation. They confirm a previous report that the adhesion molecule Hibris is required in cone cells for normal intercalation, and find that its partner Roughest also plays a role, especially in primary pigment cells. They find that Delta in the anterior cone cell signals primarily to the equatorial and polar cone cells to induce a Notch-mediated transcriptional mechanism necessary for intercalation. Finally, they argue based on computational modeling that both myosin and adhesion contribute to junctional tension, and that contraction of the primary pigment cells is necessary for cone cell intercalation.
The work is extensive and technically sound and each of the findings adds something to our understanding of the intercalation process, which occurs on a much longer timescale than most intercalations that have been studied. However, the paper does not provide very much insight into how these different mechanisms are related.

Comments for the author

1. The computational model in Fig. 6 does not seem to correspond closely to the data shown in the other figures. If myosin on cone cell contacts has only a minor role in intercalation, why is it included as an essential aspect of the model? If tensile forces in the primary pigment cells are necessary, why is intercalation disrupted only when RhoA is depleted in only one of the two pigment cells (a result that is shown as a single example and would need to be quantified) and not in the rok mutant clones shown in Fig. 1? In Fig. 1, it would be helpful to include controls to show how effectively myosin function is perturbed by Zipper[DN] and zipper RNAi. Does expressing these constructs in the primary pigment cells disrupt intercalation?
2. The model is not very clearly explained, but it seems that the adhesion values used are based on the levels of Hbs and Rst, both of which are very low at cone cell-cone cell junctions. The adhesion molecule Ncad is strongly expressed there, and it is not clear why it was not included.
3. It would increase the impact of the paper if the authors could identify which Notch transcriptional targets contribute to cone cell intercalation, and whether endocytosis is required because of its effect on Notch signaling. Again, the effects of endocytosis and Notch signaling are not incorporated into the model, making it unclear whether adding tension in the pigment cells is the only way to successfully model the intercalation process.

Reviewer 2

Advance summary and potential significance to field

This manuscript by Blackie et al. studies the mechanisms of cell intercalation in the four cone cells of the Drosophila eye. The authors identify several new mechanisms of slow intercalation (over hours) in the eye that are distinct from fast intercalation (over minutes) in other tissues. Despite a significant enrichment of MyoII at the shrinking AJ in the cone cell quartet, the authors show using rok mutant clones that cell intercalation is largely (though not completely) normal in the absence of Rok-mediated MyoII activity suggesting that the Rok-MyoII pathway does not play a major role. Using a thermo-sensitive Shibire mutant, the authors show that inhibiting endocytosis blocks multiple steps of cone cell intercalation when Shibire activity is inhibited at specific stages, a powerful approach that is made possible by the slow rate of cone cell intercalation. The authors show that Hbs and Rst localize to the cone cell-primary pigment cell boundary and the boundary between primary pigment cells. Using elegant mosaic analysis, they show that decreasing Hbs in the A and P cells or decreasing Rst in the Pl, Eq, or primary pigment cells causes intercalation defects, suggesting that differential Hbs/Rst activity is important for cone cell intercalation.

© 2021. Published by The Company of Biologists under the terms of the Creative Commons Attribution License (https://creativecommons.org/licenses/by/4.0/).

The authors also look at the effects of Notch/Delta signaling on cone cell intercalation. Based on the localization of N, Dl, Neur, and the NICD Notch activity reporter, they propose that Dl in the A cell activates N signaling in the other cone cells. Using dominant negative Mastermind to inhibit transcription downstream of Notch, and Bearded expression to inhibit Neur-dependent Dl signaling, they show that Notch activity in the Eq and Pl cells, activated by Dl in either the A or P cell, is required for cone cell intercalation. Lastly, the authors develop a computational vertex model based on measurements of Hbs and Rst localization and laser ablation studies. This model indicates that both myosin contractility and differential adhesion are needed to achieve a stable post-intercalation state, and suggests that myosin tension in the primary pigment cells surrounding the cone cells affects cone cell intercalation nonautonomously, a prediction that is borne out by preliminary experiments.

This work is very well written, extensively quantified, and the results are interpreted with appropriate caution. The authors quantify the relative contributions of four processes to slow intercalation in Drosophila cone cells, including Notch-dependent transcription, cell interactions mediated by Hibris and Roughest, endocytosis, and MyoII. This very interesting work provides insight into the mechanisms of slow junction remodeling in epithelia and will be of interest to a broad developmental biology audience. I only have a few minor comments.

Comments for the author

1. The requirement for MyoII tension in the computational model in Figure 6 appears to be inconsistent with the absence of an intercalation defect in rok2 mutant clones in Figure 1N. The SqhGFP enrichment at the central CC AJ is modest, but an enrichment over many hours could be important for intercalation. The rok2 experiment also appears to contradict the requirement for tension in primary pigment cells in the model and in the experiment in Figure 6G. Can the authors reconcile these results? Could other defects in rok2 clones obscure the requirement for myosin in primary pigment cells or in other cells of the ommatidium? Could some myosin activity remain in these clones due to Rok perdurance?

2. In the text describing Figure 2, it is not clear why the authors propose that expansion of the apical perimeter could cause the change in shape of the cone cell cluster, as opposed to other possible relationships between these measurements.

3. For the experiment showing RhoA inhibition in a primary pigment cell in Figure 6G, the genotype number of ommatidia tested, and percentage of ommatidia with arrested intercalation should be indicated. Can the authors use the rok2 clones in Figure 1 to address whether decreasing myosin activity specifically in the primary pigment cells alters intercalation?

4. Although beyond the scope of the present work, it would be interesting if the authors could use their model to test whether modulating Rst or Hbs adhesion in different cells in the model can account for the defects in Rst and Hbs mosaics.

5. The number of ommatidia should be indicated in Figure 1L and the error bars in Figure 2C, 2D, and 5E should be defined. The y-axis label in Figure 5I should be “Number of reversions”. Enfolds should be “unfolds” on page 4, line 1. Details should be “detail” on page 4, line 5. Principle should be “principal” on page 5, line 6. Figure 1M-O should be “Figure 1M-N” on page 6, line 8. Reveled should be “revealed” in the middle of page 9.

Reviewer 3

Advance summary and potential significance to field

Blackie et al. report on possible mechanisms that direct changes in cone cell topologies such that cell intercalation occurs. They examine the function of Notch/Delta signaling, endocytosis, Myosin II, and Roughest/Hibris-mediated cell adhesion. They also perform computational modeling to try and determine the mechanism required for cell intercalation in the eye. The manuscript is a confusing read, largely in part due to the fact that a clear mechanism for driving cell intercalation could not be identified. On the other hand, they carefully implicate each of the above pathways in

© 2021. Published by The Company of Biologists under the terms of the Creative Commons Attribution License (https://creativecommons.org/licenses/by/4.0/).

intercalation to varying degrees Â– it is just that how these individual pathways regulate intercalation is uncertain. Additionally, what the potential interactions between each pathways is also uncertain. For these reasons, the manuscript often reads like a series of disparate observations, and the insightfulness of the manuscript is somewhat limited, but it also represents a considerable body of work that establishes the ground state contributions of 4 pathways to cone cell intercalation, although some of the pathways have been previously implicated (for ex., Hbs in Grillo-Hill and Wolff, 2009). Analysis and statistics appears to be appropriately done throughout. More detailed comments follow below:

Comments for the author

1) The assumptions and outputs of the mathematical model do not make much sense to me (or perhaps that could be better explained?). For example, Figure 1 establishes that Myosin II has a very limited role in intercalation, and yet inputs from Myosin II localization are a major part of the modeling – does this make sense? It seems that Rst/Hbs function is a major component of intercalation, but is it not hard to reconcile that the model suggests only a 30-50% contribution? This is one of the elements that makes the paper feel like it is composed of disparate observations. It could be better to not include this data, as some of the model predictions are not tested and/or supported by experimental observations. It feels as though this was a separate set of experiments that were added in at the end of the paper.

2) The authors discuss their inclusion of the endocytosis data as a testing of mechanism that could, “couple shrinking of the A/P AJ and concomitant lengthening of the adjunct AJs.”, but this does not truly test this.
Indeed, later in the manuscript they suggest that endocytosis could be required for Notch signaling or other pathways they are studying. It is difficult to conclude whether recycling is playing a role in this system with these experiments.

3) In a similar fashion – how do the interface lengths change in the various Rst/Hbs compromised backgrounds? Do PC interfaces lengthen or is the interface coupling changed? This could help explain where Rst/Hbs function and how they are causing changes in neighbor exchange events – this would also relate it back to some of the first measurements in the paper.

4) I found the long section on Notch signaling confusing – as the authors unspooled their data I kept trying to form a model in my head of what the data could mean, only to come to the very end of the section and read that, “our results on Hbs-Rst and N signaling do not point to a simple relationship between N activity and the expression of Hbs”. It would have been less frustrating to give some indication of this earlier in the section.

5) Figure 3 would benefit from showing control (non-restrictive temp) data.

6) Do the findings shown in Figure 4 (esp. 4G) in which Eq/PI junctions do not seem to have Rst/Hbs contradict the functional requirement for Hbs in the same cells?

Minor notes:
a) The authors should better cite the intercalation literature (for example, adhesion and endocytic literature).
b) There is not a true endogenous Sqh:GFP (it is a transgene at an ectopic site). There is a Zip:GFP in which GFP is incorporated at the endogenous locus.
c) Fig 5I, typo “revertions”

First revision

<u>Author response to reviewers' comments</u>

We would like to thank both our reviewers for their insightful comments and suggestions.

© 2021. Published by The Company of Biologists under the terms of the Creative Commons Attribution License (https://creativecommons.org/licenses/by/4.0/).

Reviewer #1 (Remarks to the Author):
1. The computational model in Fig. 6 does not seem to correspond closely to the data shown in the other figures. If myosin on cone cell contacts has only a minor role in intercalation, why is it included as an essential aspect of the model? – The model is not very clearly explained, but it seems that the adhesion values used are based on the levels of Hbs and Rst, both of which are very low at cone cell-cone cell junctions. The adhesion molecule Ncad is strongly expressed there, and it is not clear why it was not included.

RE: We have modified the manuscript to better explain the basis for including MyoII in the mathematical model. In short, MyoII contractility and cell-cell adhesion are coupled in the junctional tension term of the vertex model and relative MyoII intensity measurements are typically used to parameterise junctional tension, alongside with laser ablations. This does not imply that MyoII contractility is dominant and indeed, our model predicts that this is not the case.
In this revised version of our manuscript, we have moved the mathematical model earlier (now Figure 3), right after our MyoII measurement. This allows us to better capitalise on its predictive value, instead of presenting the model in the end, as a way to integrate all our data, which was coming with the caveat that the reviewer raised here.
In addition, to address the reviewer's comment more directly, we have revisited the parameterisation of the mathematical model by taking into account intensity measurements for Ecadherin, Ncadherin, Hbs and Rst.

2. If tensile forces in the primary pigment cells are necessary, why is intercalation disrupted only when RhoA is depleted in only one of the two pigment cells (a result that is shown as a single example and would need to be quantified) and not in the rok mutant clones shown in Fig. 1?

RE: We examined 4 retinas in these shRNAi RhoA experiments, and the phenotype we show was rare (3 ommatidia in total). However, such defect is never seen in wild-type retinas.
In these experiments, we combined the Spa-Gal4 driver with Pros-Gal80 to specifically express shRNAi in the newly specified primary pigment cells. The caveat with this approach is that it does not give much time for the shRNAi to be expressed. As a consequence, even- though all primary pigment cells express the shRNAi , only a very small fraction of the ommatidia presented a complete failure in cone cell intercalation. Revisiting these experiments, we quantified the length of the AJ that forms between the polar and equatorial cone cells after intercalation and found it shorter than in the wild-type (Figure 4F). These results are in good agreement with our model that the tension in the primary pigment cell does contribute to determining the arrangement and junction lengths between the cone cells. The fact that cone cell intercalation still proceeds when tension is reduced in the primary pigment cells suggests that the system is more towards the left of the predictive model heatmap in the adhesion dominant zone.

<u>Results – Line 251:</u> 'Next to assess the second prediction of our vertex model, that if MyoII contractility dominates at the cone cell AJs, then stable intercalation would require pulling tension from the primary pigment cells, we expressed the shRNAi against RhoA specifically in the primary pigment cells (Figure 4F). We found this did not block cone cell intercalation. However, quantification of the length of the AJ that forms between the polar and equatorial cone cells after intercalation showed that this AJ was shorter (Figure 4F',F''). Specific expression of the shRNAi transgene was controlled by expressing a UAS-RFP protein (Supplementary figure 2). Thus, while regulation of the contractile actomyosin cytoskeleton in the pigment cells promotes lengthening of the AJ between the Equatorial and polar cone cells, stable intercalation is still achieved, which, put together with the predictive heatmap generated by our model (Figure 3D), suggests that this tissue lies towards the upper/left stable region where intercalation is mainly adhesion dominated.'

Our new data on tension requirement in surrounding pigment cells are in good agreement with our mathematical model.

As pointed out by the reviewer, the *rok* phenotype (using mutant clones) does not show much of a phenotype. We think this is because the MyoII regulator Genghis Khan acts alongside *rok* in retinal cells. Our *rok* clones comes with the well documented planar polarity phenotype – defects in ommatidia rotation in the plane of the tissue – confirming that Rok regulates this aspect of retinal patterning, which is thought to be due to MyoII regulation. However, it seems loss of this kinase is not sufficient to perturb the actomyosin cytoskeleton in a way that would affect cell shape. We

© 2021. Published by The Company of Biologists under the terms of the Creative Commons Attribution License (https://creativecommons.org/licenses/by/4.0/).

have recently shown MRCK (Genghis khan) is the main kinase that regulates MyoII activation in the developing photoreceptor during apical membrane morphogenesis (Zihni et al., 2017). *gek* mutant clones do not affect the other retinal cells.
However, we reasoned that *gek* could act alongside *rok* in regulating contractility in the cone and pigment cells. To test this we generated cells deficient for both *rok* and *gek*. This led to very strong defects in retinal tissue patterning, including supernumerous cone cells, which prevented us from examining cone cell intercalation (Please see figure below).

This redundancy between *rok* and *gek* is interesting, but unfortunately, it is not helping us to precisely assess the role of the actomyosin cytoskeleton during cone cell intercalation. In our revised version, we opted to remove reference to our *rok* clones as we feel our new RhoA^{N19} and shRNAi (Figure 4) are simpler to interpret and allow us to reach a stronger conclusion on the role of the actomyosin cytoskeleton during cone cell intercalation.

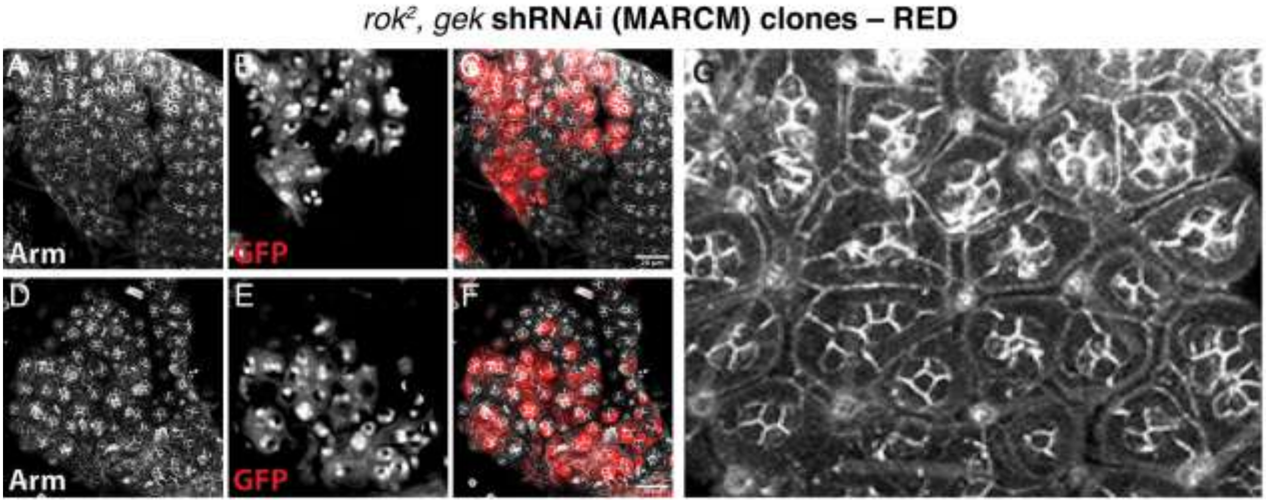

3*. In Fig. 1, it would be helpful to include controls to show how effectively myosin function is perturbed by Zipper[DN] and zipper RNAi. Does expressing these constructs in the primary pigment cells disrupt intercalation?*

RE: We have used the Zipper^{DN} before to successfully inhibit apical constriction at earlier stages of retinal development – so we know in principle this approach can work in the eye (Robertson et al., 2012). However, when expressing it in the primary pigment cells, which we know require MyoII to maintain their shape (Blackie et al., 2020), we found it does not lead to any significant phenotype. Therefore, these lens cells seem somewhat resistant to this type particular perturbation, which raises the issue that a similar 'resistance' might occur in the cone cells.

We modified this section of the manuscript accordingly.
Results – Line 239: 'Similarly, expressing a shRNAi (IR) against the heavy chain of MyoII (Zipper; *zip*) in the cone cells interfered with intercalation in less than 10% of the cases (Figure 4C). In these experiments, we cannot measure how much MyoII activity is inhibited and it is therefore possible we are underestimating the role of MyoII in cone cell intercalation'

To complement these experiments, we tried interfering with RhoA using the N19 dominant negative (Figurer 4D) and shRNAi (Figure 4E). We made use of the coin-flip system (Bosch et al., 2015) to target the cone cells using shRNAi RhoA and RhoA^{N19} (dominant negative transgene). With coin-flip, Gal4 is expressed earlier than with the Spa-Gal4 line, which allows for the transgenes expression to builds up. In these experiments the targeted cone cells relax their apical profiles, which is consistent with an inhibition of the contractile actomyosin cytoskeleton in these cells. Yet, cone cell intercalation is fine when myosin is removed from the cone cells (Figure 4D). Altogether, we therefore think we have multiple, independent evidence that MyoII contractility is minimally required for the cone cells for intercalation. We have added these results, next to the Zip shRNAi and Zipper^{DN} ones, in Figure 4.

© 2021. Published by The Company of Biologists under the terms of the Creative Commons Attribution License (https://creativecommons.org/licenses/by/4.0/).

We modified this section of the manuscript accordingly.
Results – Line 243: 'For this reason and to further probe the contractile actomyosin cytoskeleton, we also examined requirement for the MyoII activator RhoA. Expressing a dominant negative RhoA transgene (RhoA^{N19}) or shRNAi targeting this small GTPase in cone cells, led to significant relaxation of these cells, which strongly suggests inhibition of the contractile actomyosin cytoskeleton (Figure 4D-E). However, this did not affect cone cell intercalation. Therefore, taken together, our experiments indicate a minimal requirement for the contractile actomyosin cytoskeleton during cone cell intercalation.'

4. *It would increase the impact of the paper if the authors could identify which Notch transcriptional targets contribute to cone cell intercalation, and whether endocytosis is required because of its effect on Notch signaling. Again, the effects of endocytosis and Notch signaling are not incorporated into the model, making it unclear whether adding tension in the pigment cells is the only way to successfully model the intercalation process.*

RE: An obvious candidate pathway mediating N-signalling is the Rst/Hbs pathway. Notch has been shown to promote Hbs expression. For thi reason we tried rescuing the Man^{DN} phenotype in cone cell intercalation by re-introducing Hbs. However we did not see a rescue. We therefore think that N regulates the expression of multiple factors in intercalation and more work will be required to identify these pathways.

Results – Line 369: 'From these experiments, we conclude that signaling from either the A or P cell is sufficient to activate N in the Eq and Pl cells and that N activity in the Eq and Pl cone cells is critical for intercalation. This pattern of N and the requirement for Hbs in all cone cells does not suggest a simple Notch-Hbs link in this case. In addition, expressing Hbs in Pl/Eq cone cells also expressing Mam^{DN}, did not rescue the intercalation phenotype (not shown).'

Regarding a possible link between endocytosis and N activity, we agree with the reviewer that a strong possibility is that inhibiting endocytosis will affect N signalling. Indeed, our experiment manipulating Dl endocytosis (when we express Brd^{DN}) shows this is the case and we have better stressed this in the revised manuscript.

Results – Line 399: 'Another aspect of cone cell intercalation that is expected to be affected when blocking endocytosis, is the N pathway, as N-signaling requires endocytosis of Dl. To test this, we specifically targeted Dl endocytosis by over-expressing a stabilized version of Bearded (Brd) (Leviten et al., 1997), called Brd^{R} (Perez-Mockus et al., 2017). We found that expression of Brd^{R} simultaneously in the A and P cone cells led to defects in intercalation in 40% of the cases, while expressing Brd^{R} in either the A or P cell rarely led to defective intercalation (Figure 7I). Thus, next to promoting membrane removal, endocytosis is also required for N-activity in cone cell intercalation.'

Discussion – Line 491: 'Similarly, endocytosis of Dl is required to activate the Notch pathway (Schweisguth, 2004; Weinmaster and Fischer, 2011). Indeed, blocking Dl endocytosis by expressing brd^{DN} in the A/P cone cells interferes with intercalation.'

Reviewer #2 (Remarks to the Author):

1. *The requirement for MyoII tension in the computational model in Figure 6 appears to be inconsistent with the absence of an intercalation defect in rok2 mutant clones in Figure 1N. The SqhGFP enrichment at the central CC AJ is modest, but an enrichment over many hours could be important for intercalation. The rok2 experiment also appears to contradict the requirement for tension in primary pigment cells in the model and in the experiment in Figure 6G. Can the authors reconcile these results? Could other defects in rok2 clones obscure the requirement for myosin in primary pigment cells or in other cells of the ommatidium? Could some myosin activity remain in these clones due to Rok perdurance?*

RE: As discussed in the first version of the paper, we envisage the absence of phenotype in *rok*^{2} mutant clones is due to redundancies in the contractile actomyosin pathway. We think this is because the MyoII regulator Genghis Khan acts alongside Rok in retinal cells. Our rok clones comes with the well documented planar polarity phenotype – defects in ommatidia rotation in the plane of the tissue – confirming that Rok regulates this aspect of retinal patterning, which is thought to be

© 2021. Published by The Company of Biologists under the terms of the Creative Commons Attribution License (https://creativecommons.org/licenses/by/4.0/).

due to MyoII regulation. However, it seems loss of this kinase is not sufficient to perturb the actomyosin cytoskeleton in a way that would affect cell shape. We have recently shown MRCK (Genghis khan) is the main kinase that regulates MyoII activation in the developing photoreceptor during apical membrane morphogenesis (Zihni et al., 2017). *gek* mutant clones do not affect the other retinal cells. However, we reasoned that *gek* could act alongside *rok* in regulating contractility in the cone and pigment cells. To test this we generated cells deficient for both *rok* and *gek*. This led to very strong defects in retinal tissue patterning, including supernumerous cone cells, which prevented us from examining cone cell intercalation (Please see figure below).

This is interesting, but unfortunately, it is not helping us to precisely assess the role of the actomyosin cytoskeleton during cone cell intercalation. In our revised version, we opted to remove reference to our *rok* clones as we feel our new $RhoA^{N19}$ and shRNAi are simpler to interpret and allow us to reach a stronger conclusion on the role of the actomyosin cytoskeleton during cone cell intercalation.

rok², *gek* **shRNAi (MARCM) clones – RED**

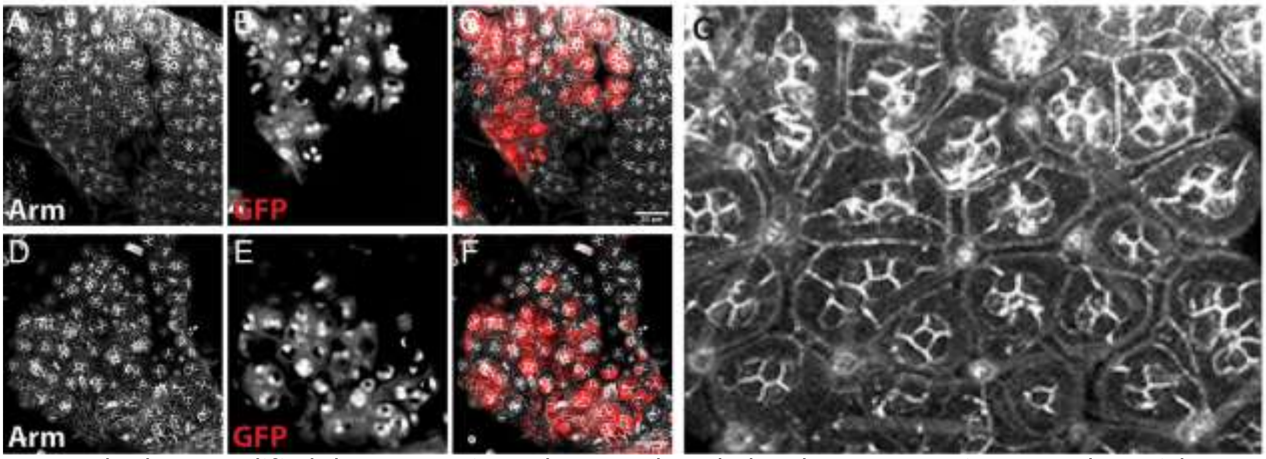

We also have modified the manuscript to better acknowledge that our genetic approaches might lead to an underestimation of the role for MyoII in cone cell intercalation.

We modified this section of the manuscript accordingly.
<u>Results – Line 239</u>: 'Similarly, expressing a shRNAi (IR) against the heavy chain of Myo-II (Zipper; *zip*) in the cone cells interfered with intercalation in less than 10% of the cases (Figure 4C). In these experiments, we cannot measure how much MyoII activity is inhibited and it is therefore possible we are underestimating the role of MyoII in cone cell intercalation'

In light of this, to complement these experiments, we tried interfering with RhoA using the N19 dominant negative (Figure 4D) and shRNAi (Figure 4E). We made use of the coin-flip system(Bosch et al., 2015) to target the cone cells using shRNAi RhoA and $RhoA^{N19}$ (dominant negative transgene). With coin-flip, Gal4 is expressed earlier than with the Spa-Gal4 line, which allows for the transgenes expression to builds up. In these experiments the targeted cone cells relax their apical profiles, which is consistent with an inhibition of the contractile actomyosin cytoskeleton in these cells. Yet, cone cell intercalation is fine when myosin is removed from the cone cells (Figure 4D). Altogether, we therefore think we have multiple, independent evidence that MyoII contractility is minimally required for the cone cells for intercalation. We have added these results, next to the Zip shRNAi and $Zipper^{DN}$ ones, in Figure 4.

We modified this section of the manuscript accordingly.
<u>Results – Line 243</u>: 'For this reason and to further probe the contractile actomyosin cytoskeleton, we also examined requirement for the MyoII activator RhoA. Expressing a dominant negative RhoA transgene ($RhoA^{N19}$) or shRNAi targeting this small GTPase in cone cells, led to significant relaxation of these cells, which strongly suggests inhibition of the contractile actomyosin cytoskeleton (Figure 4D-E). However, this did not affect cone cell intercalation. Therefore, taken together, our experiments indicate a minimal requirement for the contractile actomyosin cytoskeleton during cone cell intercalation.'

© 2021. Published by The Company of Biologists under the terms of the Creative Commons Attribution License (https://creativecommons.org/licenses/by/4.0/).

2. In the text describing Figure 2, it is not clear why the authors propose that expansion of the apical perimeter could cause the change in shape of the cone cell cluster, as opposed to other possible relationships between these measurements.

RE: We have removed this point of discussion.

3. *For the experiment showing RhoA inhibition in a primary pigment cell in Figure 6G, the genotype, number of ommatidia tested, and percentage of ommatidia with arrested intercalation should be indicated. Can the authors use the rok2 clones in Figure 1 to address whether decreasing myosin activity specifically in the primary pigment cells alters intercalation?*

RE: In these experiments, we combined the Spa-Gal4 driver with Pros-Gal80 to specifically express shRNAi in the newly specified primary pigment cells. The caveat with this approach is that it does not give much time for the siRNAi to be expressed. As a consequence, even-though all primary pigment cells express the siRNAi , only a very small fraction of the ommatidia presented a complete failure in cone cell intercalation. Revisiting these experiments, we quantified the length of the AJ that forms between the polar and equatorial cone cells after intercalation and found it shorter than in the wild type. These results are in good agreement with our model that the tension in the primary pigment cell does contribute to promoting junction lengthening between the equatorial and polar cone cells. The fact that cone cell intercalation still proceeds when tension is reduced in the primary pigment cells suggests that the system is more towards the left of the predictive model heatmap in the adhesion dominant zone. We present them in the revised version of the paper (Figure 4F)

<u>Results – Line 251: </u>'Next to assess the second prediction of our vertex model, that if MyoII contractility dominates at the cone cell AJs, then stable intercalation would require pulling tension from the primary pigment cells, we expressed the shRNAi against RhoA specifically in the primary pigment cells (Figure 4F). We found this did not block cone cell intercalation. However, quantification of the length of the AJ that forms between the polar and equatorial cone cells after intercalation showed that this AJ was shorter (Figure 4F',F''). Specific expression of the shRNAi transgene was controlled by expressing a UAS-RFP protein (Supplementary figure 2). Thus, while regulation of the contractile actomyosin cytoskeleton in the pigment cells promotes lengthening of the AJ between the Equatorial and polar cone cells, stable intercalation is still achieved, which, put together with the predictive heatmap generated by our model (Figure 3D), suggests that this tissue lies towards the upper/left stable region where intercalation is mainly adhesion dominated.'

4. *Although beyond the scope of the present work, it would be interesting if the authors could use their model to test whether modulating Rst or Hbs adhesion in different cells in the model can account for the defects in Rst and Hbs mosaics.*

RE: We agree and have now re-parameterised our model to include Hbs and Rst intensity measurements. Running simulations show that indeed Hbs/Rst adhesion promotes stable intercalation. We introduce the mathematical modelling earlier in the revised version of our manuscript, so to use highlight it's value as a predictive tools. We now take into account the levels of Hbs, Rst, Ncad and Ecad (Figure 2).

<u>Results – Section starting line 161</u>: 'Modeling cone cell intercalation predicts a predominant role for adhesion'.

5. *The number of ommatidia should be indicated in Figure 1L and the error bars in Figure 2C, 2D, and 5E should be defined. The y-axis label in Figure 5I should be "Number of reversions".*

RE: This has been done in the revised version. Fig.1L is now Fig.4B-C and the number of ommatidia scored is mentioned in the figure legend (n: 2212).
For Fig.2C, 2D (now Fig. 1F, 1G) and 5E (now Fig. 6E) the error bars have been defined in the figure legend.

6. *Enfolds should be "unfolds" on page 4, line 1. Details should be "detail" on page 4, line 5. Principle should be "principal" on page 5, line 6. Figure 1M-O should be "Figure 1M-N" on page 6,*

© 2021. Published by The Company of Biologists under the terms of the Creative Commons Attribution License (https://creativecommons.org/licenses/by/4.0/).

line 8. Revealed should be "revealed" in the middle of page 9.

RE: These typos have been corrected.

Reviewer #3 (Remarks to the Author):

1. *The assumptions and outputs of the mathematical model do not make much sense to me (or perhaps that could be better explained?). For example, Figure 1 establishes that Myosin II has a very limited role in intercalation, and yet inputs from Myosin II localization are a major part of the modeling – does this make sense? It seems that Rst/Hbs function is a major component of intercalation, but is it not hard to reconcile that the model suggests only a 30-50% contribution? This is one of the elements that makes the paper feel like it is composed of disparate observations. It could be better to not include this data, as some of the model predictions are not tested and/or supported by experimental observations. It feels as though this was a separate set of experiments that were added in at the end of the paper.*

RE: We have modified the manuscript to better explain the basis for including MyoII in the mathematical model. In short, MyoII contractility and cell-cell adhesion are coupled in the junctional tension term of the vertex model and relative MyoII intensity measurements are typically used to parameterise junctional tension, alongside with laser ablations. This does not imply that MyoII contractility is dominant and indeed, our model predicts that this is not the case.
In this revised version of our manuscript, we have moved the mathematical model much earlier (now Figure 3), right after our MyoII measurement. This allows us to better capitalise on its predictive value, instead of presenting the model in the end, as a way to integrate all our data, which was coming with the caveat that the reviewer raised here.
In addition, to address the reviewer's comment more directly, we have revisited the mathematical model by taking into account intensity measurements for Ecadherin, Ncadherin, Hbs and Rst.

2. *The authors discuss their inclusion of the endocytosis data as a testing of mechanism that could, "couple shrinking of the A/P AJ and concomitant lengthening of the adjunct AJs.", but this does not truly test this. Indeed, later in the manuscript they suggest that endocytosis could be required for Notch signaling or other pathways they are studying. It is difficult to conclude whether recycling is playing a role in this system with these experiments.*

RE: We find that blocking endocytosis leads to accumulation of membrane at the AJ shared by the cone cells (Figure 7A). We therefore conclude endocytosis regulates membrane turnover during intercalation.
<u>Result – Line 380</u>: 'Inhibiting endocytosis in the cone cells as they intercalate caused the AJs they share to become convoluted (Figure 7A). We therefore conclude that endocytosis regulates membrane turnover during cone cell intercalation.';
and
<u>Result – Line 394:</u> 'Altogether these results demonstrate that endocytosis is required for stable intercalation to proceed between the cone cells. AJ convolution is compatible with an excess of plasma membrane, which is consistent with a role for endocytosis in shedding membrane during slow intercalation.'

We also hypothesize that blocking endocytosis affects intercalation via the Notch pathway. Indeed, our experiment manipulating Dl endocytosis (when we express Brd^{DN}) show this is the case and we have better stressed this in the revised manuscript.

<u>Results – Line 399</u>: 'Another aspect of cone cell intercalation that is expected to be affected when blocking endocytosis, is the N pathway, as N-signaling requires endocytosis of Dl. To test this, we specifically targeted Dl endocytosis by over-expressing a stabilized version of Bearded (Brd) (Leviten et al., 1997), called Brd^{R} (Perez-Mockus et al., 2017). We found that expression of Brd^{R} simultaneously in the A and P cone cells led to defects in intercalation in 40% of the cases, while expressing Brd^{R} in either the A or P cell rarely led to defective intercalation (Figure 7I). Thus, next to promoting membrane removal, endocytosis is also required for N-activity in cone cell intercalation.'

<u>Discussion – Line 491</u>: 'Similarly, endocytosis of Dl is required to activate the Notch pathway (Schweisguth, 2004; Weinmaster and Fischer, 2011). Indeed, blocking Dl endocytosis by expressing brd^{DN} in the A/P cone cells interferes with intercalation.'

© 2021. Published by The Company of Biologists under the terms of the Creative Commons Attribution License (https://creativecommons.org/licenses/by/4.0/).

3. In a similar fashion – how do the interface lengths change in the various Rst/Hbs compromised backgrounds? Do PC interfaces lengthen or is the interface coupling changed? This could help explain where Rst/Hbs function and how they are causing changes in neighbor exchange events – this would also relate it back to some of the first measurements in the paper.

RE: Following this comment, we quantified the length of the cone-pigment interfaces. We found inhibiting *rst/hbs* affects these lengths, however, we could not infer from this which are the cell types where Rst and Hbs function, beyond what we had inferred from our mosaics.

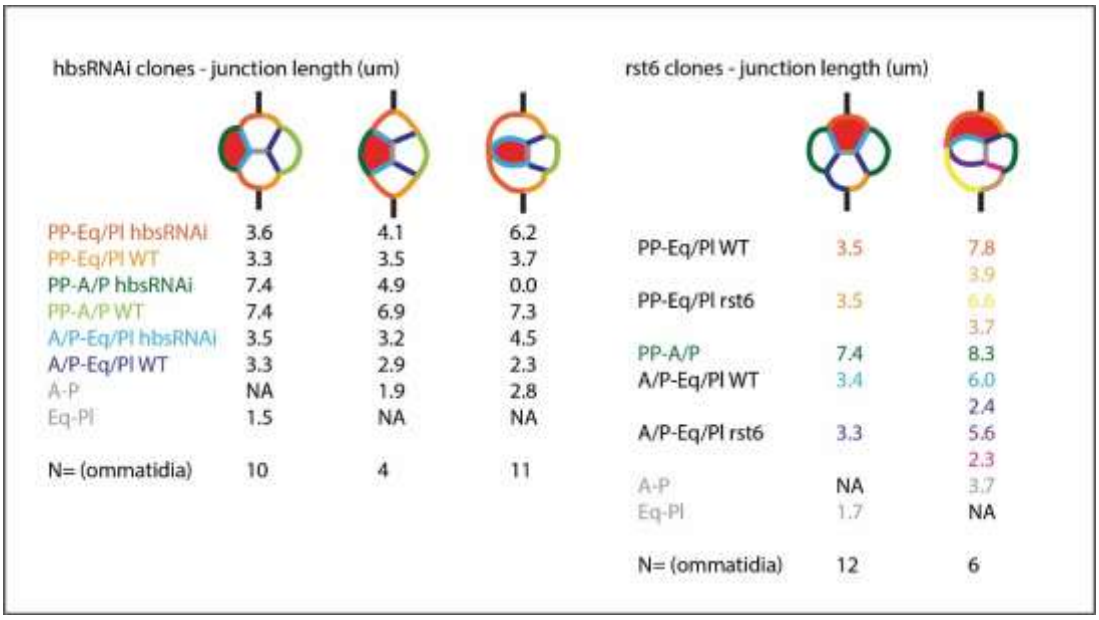

4. I found the long section on Notch signaling confusing – as the authors unspooled their data I kept trying to form a model in my head of what the data could mean, only to come to the very end of the section and read that, "our results on Hbs-Rst and N signaling do not point to a simple relationship between N activity and the expression of Hbs". It would have been less frustrating to give some indication of this earlier in the section.

RE: To ameliorate this section we have broken it up into two distinct paragraphs: ***i)*** a first section describing which cells present Dl and which present N/N-activity, and ***ii)*** a second section to assess what role this system might play during cone cell intercalation, which leads to the conclusion that signaling from either the A or P cell is sufficient to activate N in the Eq and Pl cells and that N activity in these two cells is critical for intercalation.

5. Figure 3 would benefit from showing control (non-restrictive temp) data.
RE: These data are now figure 7. A control for the Gal4 with 'mock' UAS is shown in figure 4C at the non-restrictive temperature. We routinely raise animals at different temperatures, including 31 degrees and have never observed this impact on cone cell intercalation. We are therefore confident the phenotype is linked to the *Shi*^{ts} transgene.

6. Do the findings shown in Figure 4 (esp. 4G) in which Eq/Pl junctions do not seem to have Rst/Hbs contradict the functional requirement for Hbs in the same cells?
RE: In this drawing (4G, now 5G), we meant to show where Rst and Hst are detected. This includes the AJs shared between the cone and primary pigment cells. However, considering the polar and equatorial cone cells, the detectable signal is restricted to a small portion of the membrane. We have extended this domain to make it clearer that we detect Hbs and Rst in all 4 cone cells.

- The authors should better cite the intercalation literature (for example, adhesion and endocytic literature).

RE: In our revised version we are including a discussion on Ecad endocytosis , which includes

© 2021. Published by The Company of Biologists under the terms of the Creative Commons Attribution License (https://creativecommons.org/licenses/by/4.0/).

reference to its role in intercalation in the fly embryo.

Discussion – Line 494: 'Endocytosis is also likely to be required to control the turnover of Ecad/Ncad in the cone cells and promote plasma membrane removal during AJ shrinkage. For instance, as these cells intercalate Ecad becomes depleted from the AJ between the cone cells and Ecad overexpression in the Eq/Pl cone cells blocks intercalation (Carthew, 2005). It is possible that endocytosis promotes this inhibition. Consistent with this type of model, similar regulation of Ecad endocytosis takes place in the fly embryo during intercalation to shrink AJs (Levayer et al., 2011).'

- There is not a true endogenous Sqh:GFP (it is a transgene at an ectopic site). There is a Zip:GFP in which GFP is incorporated at the endogenous locus.
- Fig 5I, typo "revertions"

RE: We have corrected these mistakes

Blackie, L., Walther, R. F., Staddon, M. F., Banerjee, S. and Pichaud, F. (2020). Cell-type- specific mechanical response and myosin dynamics during retinal lens development in Drosophila. *Mol Biol Cell* **31**, 1355-1369.
Bosch, J. A., Tran, N. H. and Hariharan, I. K. (2015). CoinFLP: a system for efficient mosaic screening and for visualizing clonal boundaries in Drosophila. *Development* **142**, 597- 606.
Carthew, R. W. (2005). Adhesion proteins and the control of cell shape. *Curr Opin Genet Dev* **15**, 358-363.
Levayer, R., Pelissier-Monier, A. and Lecuit, T. (2011). Spatial regulation of Dia and Myosin-II by RhoGEF2 controls initiation of E-cadherin endocytosis during epithelial morphogenesis. *Nat Cell Biol* **13**, 529-540.
Leviten, M. W., Lai, E. C. and Posakony, J. W. (1997). The Drosophila gene Bearded encodes a novel small protein and shares 3' UTR sequence motifs with multiple Enhancer of split complex genes. *Development* **124**, 4039-4051.
Perez-Mockus, G., Roca, V., Mazouni, K. and Schweisguth, F. (2017). Neuralized regulates Crumbs endocytosis and epithelium morphogenesis via specific Stardust isoforms. *J Cell Biol* **216**, 1405-1420.
Robertson, F., Pinal, N., Fichelson, P. and Pichaud, F. (2012). Atonal and EGFR signalling orchestrate rok- and Drak-dependent adherens junction remodelling during ommatidia morphogenesis. *Development* **139**, 3432-3441.
Schweisguth, F. (2004). Regulation of notch signaling activity. *Curr Biol* **14**, R129-138.
Weinmaster, G. and Fischer, J. A. (2011). Notch ligand ubiquitylation: what is it good for? *Dev Cell* **21**, 134-144.
Zihni, C., Vlassaks, E., Terry, S., Carlton, J., Leung, T. K. C., Olson, M., Pichaud, F., Balda, M. S. and Matter, K. (2017). An apical MRCK-driven morphogenetic pathway controls epithelial polarity. *Nat Cell Biol* **19**, 1049-1060.

Second decision letter

MS ID#: DEVELOP/2020/197301

MS TITLE: A combination of Notch signaling, preferential adhesion and endocytosis induces a slow mode of cell intercalation in the Drosophila retina.

AUTHORS: Laura Blackie, Melda Tozluoglu, Mateusz Trylinski, Rhian Faye Walther, Francois Schweisguth, Yanlan Mao, and Franck Pichaud
ARTICLE TYPE: Research Article

I am happy to tell you that your manuscript has been accepted for publication in Development, pending our standard ethics checks.

© 2021. Published by The Company of Biologists under the terms of the Creative Commons Attribution License (https://creativecommons.org/licenses/by/4.0/).

<u>Reviewer 1</u>

Advance summary and potential significance to field

In this paper, Blackie et al. identify several mechanisms that contribute to junctional remodeling during neighbor exchanges between the four cone cells in the Drosophila retina, a complex epithelium composed of multiple distinct cell types. The authors show that myosin is enriched at the shrinking junction between the polar and equatorial cone cells, but perturbing myosin function has only a minor effect on cell intercalation. Shrinkage of this junction correlates with expansion of the adjoining junctions and expansion of the 4-cell cluster along the same dimension. They argue based on computational modeling that adhesion plays a larger role than myosin contractility in successful cone cell intercalation, and confirm that myosin is not essential using new strategies to disrupt it. They confirm a previous report that the adhesion molecule Hibris is required in cone cells for normal intercalation, and find that its partner Roughest also plays a role, especially in primary pigment cells. They find that Delta in the anterior cone cell signals primarily to the equatorial and polar cone cells to induce a Notch-mediated transcriptional mechanism necessary for intercalation. Finally, they show that blocking endocytosis prevents normal junction shrinkage and intercalation.
The work is extensive and technically sound, and adds to our understanding of the intercalation process, which occurs on a much longer timescale than most cell intercalations that have been studied. The new results strengthen the paper by showing that unlike these faster intercalations, cone cell intercalation is not primarily mediated by myosin contractility.

Comments for the author

The authors have responded well to the criticisms by adding new data and revising their model. The new organization of the paper also gives it a more logical flow and works better to put each result into context.

<u>Reviewer 2</u>

Advance summary and potential significance to field

The excellent revised paper is significantly improved and the authors have addressed all of my comments. The reorganization of the modeling results was very helpful in improving the flow of the paper and clarifying the contributions of the different processes studied. This work represents a novel and important advance in understanding the mechanisms of slow intercalation in epithelial cells.

Comments for the author

I have no further comments and I strongly support publication of this paper.

<u>Reviewer 3</u>

Advance summary and potential significance to field

Blackie et al. report on possible mechanisms that direct changes in cone cell topologies such that cell intercalation occurs. They examine the function of Notch/Delta signaling, endocytosis, Myosin II, and Roughest/Hibris-mediated cell adhesion. They also perform computational modeling to try and determine the mechanism required for cell intercalation in the eye, which is an interesting alternate system in that intercalation occurs over much slower timeframes than in other tissues in which intercalation is classically studied.

Comments for the author

The revised manuscript is improved, and better delineates some of the caveats of the approaches and ties together disparate observations in an improved fashion. I believe it is appropriate for publication in Development. Two minor suggestions:

© 2021. Published by The Company of Biologists under the terms of the Creative Commons Attribution License (https://creativecommons.org/licenses/by/4.0/).

1) At line 163, the authors say that, "Altogether, these results suggest that slow cell intercalation in the eye lens is not primarily driven by MyoII contractility." Given that this is solely based on localization data (at this point of the manuscript) of an overexpression construct, and with no functional data, I would further qualify this statement by saying, "suggests that slow cell intercalation in the eye lens MAY not be primarily driven by MyoII contractility." or "suggests a possibility that that slow cell intercalation in the eye lens is not primarily driven by MyoII contractility." In reading the statement the first time through I had a reaction that this was strong for the described data.

2) Line 413, awkward phrasing, "Thus, next to promoting membrane removal, endocytosis is also required for N-activity in cone cell intercalation." Better phrasing would be, "Thus, along with promoting membrane removal, endocytosis is also required for N-activity in cone cell intercalation." (or other alternate phrasing)

© 2021. Published by The Company of Biologists under the terms of the Creative Commons Attribution License (https://creativecommons.org/licenses/by/4.0/).
